# Modularly-Assembled Smart Microneedle Platform for Machine Learning-Driven Personalized Health Monitoring

**DOI:** 10.1007/s40820-026-02095-x

**Published:** 2026-02-09

**Authors:** Hongyi Sun, Lechen Chen, Tao Wang, Zhuoheng Li, Yi Shi, Wen Lv, Zhi Yang, Fuzhen Xuan, Min Zhang, Guoyue Shi

**Affiliations:** 1 School of Chemistry and Molecular Engineering, East China Normal University, Shanghai, 200241 People’s Republic of China; 2 WuHu Hospital, East China Normal University (The Second People’s Hospital of WuHu), Wuhu, 241000 People’s Republic of China; 3 National Key Laboratory of Advanced Micro and Nano Manufacture Technology, School of Electronic Information and Electrical Engineering, Shanghai Jiao Tong University, Shanghai, 200240 People’s Republic of China; 4 Shanghai Key Laboratory of Intelligent Sensing and Detection Technology, School of Mechanical and Power Engineering, East China University of Science and Technology, Shanghai, 200237 People’s Republic of China

**Keywords:** Microneedle, Multiplexed sensing, Flexible patch, Machine learning, Personalized health

## Abstract

Given the inherent complexity of metabolic pathways and disease-associated agents, next-generation healthcare necessitates wearable, non-invasive, and customized approaches to continuously monitor a broad spectrum of physiologically relevant biomarkers for personalized health management. Moreover, existing data-based analytical strategies remain inadequate for delivering quantitative and predictive evaluations of health status in real-life settings. Here, we report an electronic multiplexed microneedle-based biosensor patch (eMPatch) that enables real-time, minimally invasive monitoring of key metabolic biomarkers in interstitial fluid, including glucose, uric acid, cholesterol, sodium, potassium, and pH. By integrating modular microneedle (MN) sensors into a skin-interfaced flexible platform, the eMPatch achieves robust mechanical stability and seamless skin conformity, thereby ensuring reliable and continuous sensing within the dermal space. In vivo validation in animal models under metabolic intervention highlights the strong capability of the eMPatch for real-time physiological tracking across diverse daily activities. Implemented with a machine learning algorithm, the eMPatch enables automatic feature extraction and multi-task health assessment, achieving a classification accuracy of 0.996 in distinguishing normal and diet-induced metabolic disorder for health condition identification and an R^2^ score of 0.977 for the corresponding degree evaluation. This study highlights the potential of the MN-integrated, machine learning-enhanced biosensing platform toward personalized health management.

**Supplementary Information:**

The online version contains supplementary material available at 10.1007/s40820-026-02095-x.

## Highlights

- A skin-interfaced microneedle patch simultaneously and continuously measures six metabolic biomarkers from dermal interstitial fluid—glucose, uric acid, cholesterol, sodium, potassium, and pH.
- Modular microneedle units assembled on a compliant polystyrene-isoprene-polystyrene substrate offer mechanical robustness and excellent flexibility, enabling seamless adhesion, stable skin-sensor coupling, and user-specific configuration, which delivers durable, conformal wear with high signal fidelity in daily use.
- An end-to-end personalized health evaluation system: high-dimensional multiplexed signals are processed by an optimized machine-learning pipeline to quantify and predict metabolic responses to daily behaviors, supporting personalized guidance (e.g., postprandial control, electrolyte balance).

**Supplementary Information:**

The online version contains supplementary material available at 10.1007/s40820-026-02095-x.

## Graphical abstract

**Figure:**
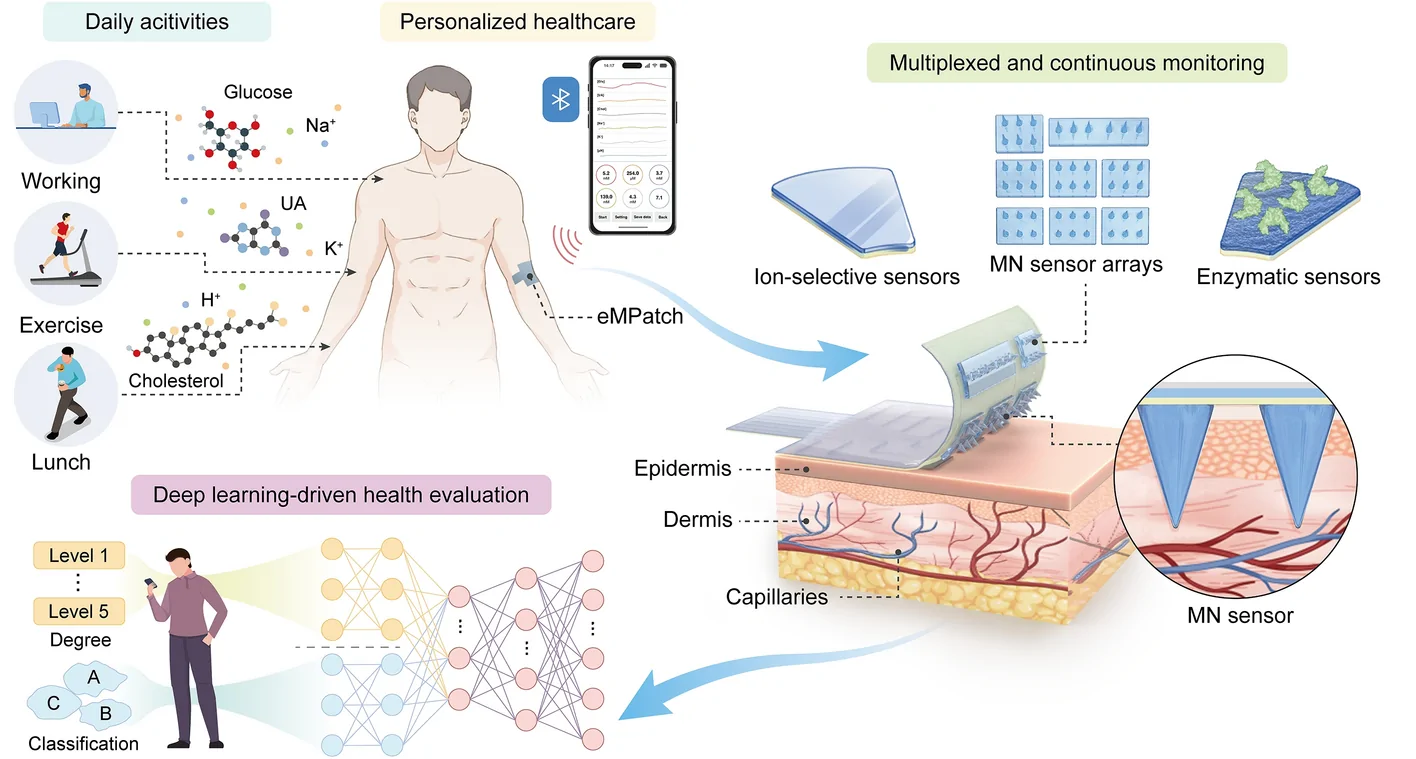

## Introduction

**Fig. 1:**
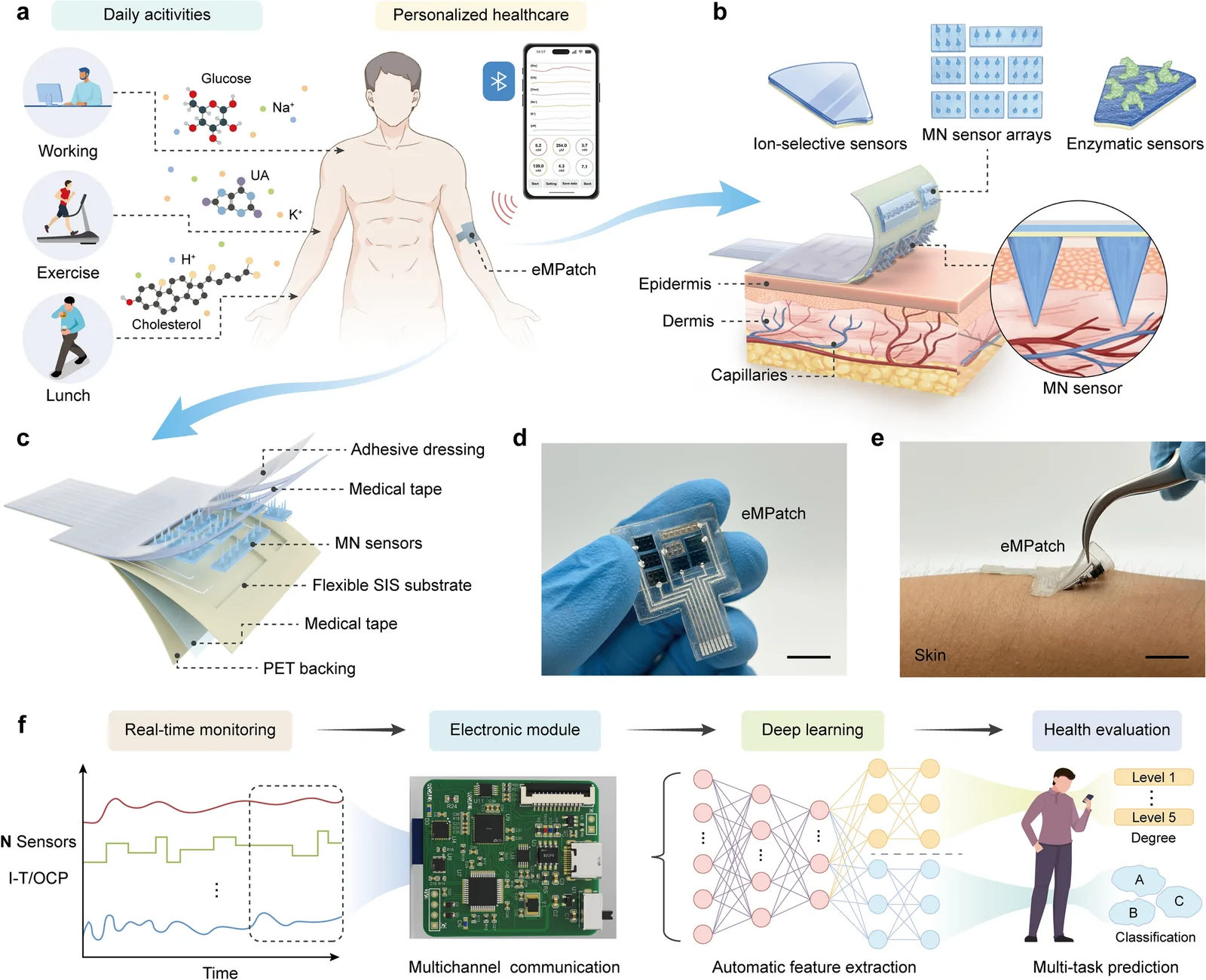
eMPatch for personalized health management. **a** Schematic showing the role of the eMPatch for personalized healthcare during daily activities. **b** Schematic illustrating the modular configuration of the eMPatch with arrays of ion-selective and enzymatic sensors and illustration of the eMPatch on the skin, exhibiting the minimally invasive approach of MN in the dermis. **c** Schematic displaying the multi-layered structure of the eMPatch. **d-e** Photographs of the eMPatch. Scale bar, 1 cm. **f** Schematic of deep learning-driven data processing for personalized health management

By harnessing the rapid advancements of wearable biosensing technologies, wireless electronics, miniaturized system integration, and data-driven analytical strategies, next-generation personalized healthcare devices are emerging as transformative paradigm in medical diagnostics, enabling real-time, non-invasive metabolic profiling and precision assessment at the individual level [1–3]. In contrast to conventional gold-standard blood and urine analyses that are inherently invasive, time-consuming, resource-intensive, and limited in temporal resolution, skin-interfaced wearable biosensors continuously transduce dynamic metabolic fluctuations into quantifiable biomedical signals for understanding key physiological parameters related to the wearer’s health status (e.g., metabolic dysregulation, stress) and facilitate proactive disease management (e.g., chronic kidney disease, diabetes) with minimal clinical intervention (Fig. 1a) [4–6].

To this end, wearable biosensors have been widely integrated into healthcare applications to enable continuous, on-body monitoring of physiologically relevant biomarkers in alternative biofluids, such as sweat, tears, saliva, and interstitial fluid (ISF) [7–9]. However, epidermal biosensing platforms face intrinsic limitations, often requiring sophisticated microfluidic configurations, anti-interference strategies, and intricate sample collection or dilution processes to ensure analytical reliability [10, 11]. These challenges collectively hinder the practical translations for long-term health surveillance and clinical decision-making. Among the biofluid-based sensing modalities, dermal ISF—defined as a peripheral biofluid surrounding cellular and tissue matrices—provides a metabolically informative medium that exhibits strong correlations with blood biomarkers owing to the continuous transcapillary exchange with systemic circulation [12, 13]. Instead of surface sampling, direct ISF investigation circumvents the inherent drawbacks of non-invasive epidermal biosensing, including ambient contamination, sampling inconsistency, and temporal delays [14–16]. Consequently, ISF-oriented sensing is emerging as a compelling avenue for real-time, high-fidelity physiological monitoring.

Microneedle (MN) technology, characterized by its mechanically sharp, robust, yet minimally invasive needle-like architecture, ensures efficient skin penetration without bleeding or tissue damage, allowing direct, continuous, and real-time ISF analysis [17–19]. The unique capability of MN can be greatly enhanced by integrating on-tip electrochemical sensing with noble metal-sputtered solid interface to track clinically significant, blood-correlated biomarkers with high specificity and accuracy, positioning MN-based biosensors as an innovative solution capable of bridging the long-standing gap between laboratory-based diagnostics and decentralized evaluation in personalized medicine and digital healthcare [20–23]. However, existing MN-based biosensors predominantly focus on single-biomarker detection (e.g., glucose), resulting in limited metabolic insights and incomplete health evaluations [24–26]; the absence of a universal and adaptable fabrication strategy constrain the development of multiplexed MN architecture capable of simultaneous multi-biomarker monitoring; seamless integration of rigid MN components with soft, skin-compatible substrates remains technically challenging, often compromising wearability, comfort, and long-term operational stability [27–29]; the lack of robust data analytics results in suboptimal extraction, classification, and prediction of metabolic trends and thereby the clinical utility in personalized health management [30, 31]. Collectively, there is a strong desire for a miniaturized, multiplexed, and flexible MN-based biosensing platform that not only enables comprehensive metabolic monitoring but also integrates algorithm-driven interpretation and predictive health analysis in precision healthcare, yet such a prototype remains largely unfulfilled.

Here, we present a skin-interfaced flexible electronic multiplexed MN-based biosensor patch (eMPatch) capable of simultaneous monitoring of six metabolic biomarkers in real-time, thereby enabling holistic and dynamic profiling of metabolic variations during daily activities. As a proof of concept, the MNs were fabricated and assembled into modular sensing units that allow personalized sensor configurations tailored to individual health needs. Bio-recognition elements were selectively functionalized onto the MNs to enable multiplexed detection of key metabolic biomarkers—including glucose, uric acid (UA), cholesterol, sodium (Na⁺), potassium (K⁺), and pH, which are pivotal metabolic indicators that can collectively reflect metabolic status and electrolyte homeostasis. Simultaneous monitoring of these interdependent biomarkers enables a comprehensive understanding of systemic physiological health and its dynamic variations during daily activities (Fig. 1b). Engineered with a mechanically compliant yet durable polystyrene-block-polyisoprene-block-polystyrene (SIS) substrate, the eMPatch achieved seamless skin adhesion and stable sensor-skin interactions, which greatly enhanced the wearability and signal fidelity (Fig. 1c, e). Integrating wearable MN-based biosensors with artificial intelligence algorithms represented a transformative pathway toward data-driven personalized health management. To this end, the high-dimensional multiplexed datasets acquired from the eMPatch were analyzed through an optimized machine learning (ML) framework, enabling quantitative and predictive assessment of metabolic health status with exceptional accuracy and reliability (Fig. 1f).

## Experimental Section

### Chemicals and Materials

Polystyrene (PS), potassium ferricyanide (K_3_Fe(CN)_6_), potassium ferrocyanide (K_4_Fe(CN)_6_), iron (III) chloride (FeCl_3_), hydrochloric acid (HCl), 3,4-ethylenedioxythiophene (EDOT), poly(4-styrenesulfonic acid) (PSS), uricase (UOx) (20 U mg^−1^), cholesterol oxidase (ChOx) (≥ 10 U mg^−1^), sodium ionophore X, sodium tetrakis[3,5-bis(trifluoromethyl)phenyl] borate (Na-TFPB), valinomycin, bis(2-ethylehexyl) sebacate (DOS), polyvinyl chloride (PVC), aniline, polyvinyl butyral (PVB), tetrahydrofuran (THF), D-glucose monohydrate, uric acid (UA), cholesterol, sodium chloride (NaCl), potassium chloride (KCl), calcium chloride (CaCl_2_), magnesium chloride (MgCl_2_), L-ascorbic acid (AA), L-histidine, L-tryptophan, acetylsalicylic acid (aspirin), 4-acetamidophenol, potassium oxonate (PO), hypoxanthine (HX), sodium carboxymethyl cellulose (CMC), methanol, toluene, N,N-dimethylformamide (DMF), Triton X-100, and phosphate buffer solution (PBS) (1 × , pH 7.4) were purchased from Adamas-beta. Polystyrene-block-polyisoprene-block-polystyrene (styrene 17 wt%), poly(ethylene glycol) diglycidyl ether (PEGDE), and chitosan (medium molecular weight) were purchased from Macklin Biochemical. Glucose oxidase (GOx) from Aspergillus niger (> 180 U mg^−1^) and sodium tetraphenylborate were purchased from Sigma-Aldrich. The artificial interstitial fluid (aISF) was prepared according to our previous study [32]. PDMS (Sylgard 184) was purchased from Dow Corning. Medical tapes (1587) and Tegaderm (1624W) were purchased from 3M. Polyethylene terephthalate (PET) films (thickness: 0.1 mm) were purchased from Ocan Polymer. Silver ink was purchased from Julong Electronic Technology. Standard mouse diet (AIN-93 M) and diet-induced obesity (DIO) diet (SFD010) were purchased from SPF Biotechnology. Commercial colorimetric assay kits were acquired from Nanjing Jiancheng Bioengineering Institute.

### Fabrication of MN

PS-based MN was fabricated with the micro-molding technique. A PDMS mold was prepared by mixing PDMS elastomer with a curing agent in a 9:1 ratio and stirring thoroughly for 20 min. The mixture was degassed in a vacuum oven for 15 min and then cured at 80 °C for 2 h. The layout of the MN was designed using AutoCAD software and carved onto the PDMS mold by a 25 W CO_2_ laser platform (Dahong Laser) using drill mode. The depth and radius of the laser-drilled holes were controlled by the parameters of the laser. The optimized parameters were a power of 2 W, speed of 100 mm s^−1^, and pitch of 0.05 mm. The as-fabricated PDMS mold was immersed in a PS solution (300 mg mL^−1^ in DMF) and centrifuged at 5000 rpm for 5 min. The filled mold was dried on a heating plate at 80 °C for 12 h. The resulting MN consisted of 2 × 3 microneedles with a length of 1000 μm and a diameter of 300 μm. The multiplexed sensing relies on a group of enzymatic sensors composed of three enzymatic working electrodes (WE), another group of three ion-selective working electrodes, one shared CE made of Cr/Au, and one shared Ag/AgCl RE. For WE and CE, a thin layer of Cr (~ 10 nm) was first coated onto the MN surface by magnetron sputtering at 10 mA for 20 s, followed by Au (~ 150 nm) sputtering at 25 mA for 120 s (GVC-2000, Ion Beam). Then, PEDOT: PSS was introduced to WE by electropolymerization (1040c, CH Instruments) to increase electrochemical surface area and enhance sensitivity. In brief, the Au-sputtered MN was immersed in a 0.2 wt% EDOT and 4 wt% PSS aqueous solution while applying cyclic voltammetry from 0.2 to 0.9 V at a scan rate of 50 mV s^−1^ for 10 cycles. The MN was then dried and cured at 80 °C for 1 h and chilled at 4 °C for further experiments. For RE, 10 μL of 0.05 M FeCl_3_ solution was drop-casted onto the Cr/Ag coated MN (20 mA, 90 s for Ag sputtering) for 30 s, and then rinsed with deionized water for three times, followed by drop-casting of a cocktail solution containing 50 mg NaCl, 79.1 mg PVB in 1 mL methanol for electrode protection.

### Preparation of Enzymatic Sensors

To prepare glucose and cholesterol sensors, a thin mediator layer of Prussian Blue (PB) was electrodeposited by cyclic voltammetry from − 0.2 to 0.4 V at a scan rate of 20 mV s^−1^ for 15 cycles in a fresh solution containing 2.5 mM FeCl_3_, 2.5 mM K_3_Fe(CN)_6_, 0.1 M KCl in 0.1 M HCl. The UA sensor was prepared in the same process for 5 cycles. The PB-modified MN was washed with deionized water and dried, followed by drop-casting of 10 μL enzyme cocktails onto the corresponding WE. The enzyme cocktails were prepared by mixing enzyme solutions (GOx 10 mg mL^−1^, UOx 20 mg mL^−1^, and ChOx 10 mg mL^−1^) with chitosan (1 wt% in 1% acetic acid) in an optimized ratio of 1:1. After drying, 10 μL of 1% PEGDE was drop-coated for enzyme crosslinking and protection.

### Preparation of Ion-Selective Sensors

To prepare ion-sensitive sensors, ion-selective membrane cocktails were made as follows: Na^+^ selective membrane consisted of 1 mg sodium ionophore X, 0.55 mg Na-TFPB, 33 mg PVC, and 65.45 mg DOS, which were dissolved in 660 μL THF. The K^+^ selective membrane consisted of 2 mg valinomycin, 0.55 mg sodium tetraphenylborate, 33 mg PVC, and 65.45 mg DOS, which were dissolved in 660 μL THF. Then, 10 μL of the ion-selective membrane cocktails was drop-casted onto the corresponding PEDOT:PSS-modified WE and left for drying. To prepare the pH sensor, PANI was electropolymerized onto WE by scanning cyclic voltammetry from − 0.2 to 1.0 V for 30 cycles at a scan rate of 100 mV s^−1^ using a 0.1 M aniline solution in 0.1 M HCl.

### Morphology Characterization of the MN Sensors

The morphology of the MN and modified components was characterized using scanning electron microscopy (SEM, Sigma 300, ZEISS). The element composition of the as-prepared MN sensor was determined by energy-dispersive X-ray spectroscopy (EDS) (Xplore 30, Oxford).

### Biocompatible Characterization of the MN Sensors

Mouse fibroblast cells L929 (Princella) were cultured in DMEM culture media added with 10% FBS and 1% penicillin–streptomycin at 37 °C and under 5% CO_2_ atmosphere. After cell proliferation, the L929 cells were cultured in a 24-well plate. The MN sensor was rinsed with 75% ethanol for three times and then placed into the 24-well plate for 24 or 48 h. A Cell Counting Kit-8 (CCK-8, Beyotime) was used to determine cell viability. The absorbance was measured at 450 nm using a microplate reader (Infinite M200 pro, Tecan). The morphology of cells was determined using a Live/Dead staining assay kit (Beyotime). The fluorescent images were captured by a biological inverted microscope (BDS400-FL, CNOptec).

### Fabrication and Assembly of the Flexible eMPatch

To fabricate an elastic SIS substrate, 200 mg mL^−1^ SIS solution in toluene was evenly spin-coated on a Si wafer at a speed of 3000 rpm for 60 s (WS-650–23, Laurell Technology) and cured at 80 °C for 2 h. The resulting SIS substrate with a thickness of about 0.45 mm was then cut and carved with serial 3.5 mm × 5 mm × 0.2 mm grooves, of which the layout was designed by AutoCAD software. Modified MN sensors, CE, and RE were fixed into the laser-carved grooves using an SIS solution. A laser-patterned PET tape was then attached to the SIS substrate as the printing mask. Stretchable silver ink composed of silver ink and SIS in a weight ratio of 4:1, mixed by a planetary centrifugal mixer (MSK-PVC-300, Kejing Star Technology) at a speed of 1500 rpm for 10 min, was screen printed onto the pre-patterned PET mask to form stretchable interconnects, which were connected to the fixed MNs using silver ink. After being cured at 80 °C for 1 h, the PET mask was removed and a double-sided medical tape (70–80 µm) was aligned onto the SIS substrate, followed by a layer of Tegaderm adhesive dressing (40–50 µm). Both the medical tape and Tegaderm were patterned using laser-cutting to selectively expose only the MN tips, while the surrounding baseplate was fully covered by the adhesive layer to ensure insulation and structural stability during skin attachment. Finally, the sensing area was attached with a thin layer of PET backing (0.1 mm) to support the MN during physical deformation. The optimized working parameters for the laser platform are listed in Table S1.

### In Vitro Electrochemical Characterization

Electrochemical experiments, including chronoamperometry, OCP, and CV, were performed using a multichannel electrochemical workstation (1040c, CH Instruments). Electrochemical impedance spectroscopy (EIS) was operated using CHI 660e. For the enzymatic MN sensors, chronoamperometric measurements were performed at a potential of -0.1 V in PBS (pH = 7.4) with corresponding target analytes, including glucose ranging from 0 to 20 mM, UA ranging from 0 to 1000 μM, and cholesterol ranging from 0 to 10 mM. For the ion-selective sensors, OCP tests were performed with analytes of NaCl ranging from 5 to 160 mM, and KCl ranging from 1 to 32 mM. Na_2_HPO_4_/citric acid buffer solutions were adjusted to standard pH from 3 to 8 for the pH sensor. Calibration curves were obtained from the correlation between the current/potential readouts and corresponding concentrations. Commercial colorimetric assay kits and a pH meter (PHS-3CB, Yueping) were used to validate the accuracy of the MN sensors in the aISF. Target analytes with high concentration were diluted for colorimetric detection.

To evaluate the selectivity of each sensor, commonly found interferences in ISF were added stepwise, followed by serial addition of target analytes. The selectivity of enzymatic sensors was tested using 300 μM UA, 300 μM AA, 5 mM NaCl_2_, 5 mM KCl, 500 μM histidine, 500 μM tryptophan, 100 μM aspirin, 100 μM acetaminophen, 5 mM glucose, 5 mM lactate, and 5 mM cholesterol. For the ion-selective sensors, 5 mM NaCl, 5 mM KCl, 2 mM MgCl_2_, 4 mM CaCl_2_, 5 mM glucose, 300 μM UA, and 5 mM cholesterol were used as interferents. The short-term stability was investigated by repetitive measurements of step-up concentrations of target analytes using the same one or a batch of three sensors. The continuous stability was investigated by monitoring the electrochemical responses of all sensing channels over a 120-min measurement period. The shelf-life stability was explored by recording the electrochemical performance of each sensor once a day for 14 days, during which the sensors were stored at 4 °C. The batch-to-batch reproducibility was evaluated by measuring the electrochemical signals of sensors from five batches (eight independently fabricated sensors for each batch) toward corresponding analytes within their physiological ranges. All selectivity and stability experiments were conducted in the aISF solution.

### Design and Integration of the Electronic System

The electrochemical electronic system was designed by Altium Designer software. The hardware layout comprises a microcontroller unit (MCU), an electrochemical analog front end (AFE), an external ADC chip (ADS1115), a channel selector, and a Bluetooth communication module. The low-power STM32L431 MCU, based on the ARM Cortex-M4 core, manages data acquisition, command processing, and task scheduling. The electrochemical AFE (AD5941) utilizes the serial peripheral interface (SPI) protocol for bidirectional communication with the MCU, enabling precise control over measurement modes and parameter configurations through register manipulation. External interrupts from the front end trigger the MCU to retrieve measurement results. The ADC chip communicates with the MCU via the inter-integrated circuit (I2C) protocol, ensuring reliable and high-precision acquisition of OCP signals. The channel selector features a single-pole eight-throw (1P8T) multiplexer (AD1408) and a customized adapter board connected to the mainboard via a 12-pin FPC cable. The system supports multichannel chronoamperometric and potentiometric measurements. These channels operate concurrently, with the OCP channels directly interfacing with the ADC chip for independent measurement, enabling simultaneous collection of data across all channels. Wireless communication is facilitated by a low-power Bluetooth Low Energy (BLE) module (CC2540), which pairs with a custom-designed JavaScript Object Notation (JSON)-based data exchange protocol to ensure efficient and reliable transmission of real-time data and commands. The system is powered by a 300 mAh lithium battery, supported by a USB Type-C compatible charging circuit, offering up to six hours of continuous operation.

The device operates in a single mode, simultaneously performing measurements across three chronoamperometric measurement channels and three OCP channels. The firmware architecture is based on the open-source FreeRTOS platform, enabling real-time multitasking for functions, such as electrochemical measurements, command processing, and data communication. Data acquisition is performed at an adjustable rate by the electrochemical AFE, with the MCU retrieving data via the SPI protocol upon external interrupts. Potentiometric signals are independently acquired through the ADC chip and transmitted to the MCU via I2C. Commands are parsed and queued for execution, with results packaged into JSON responses marked with statuses, such as success or error. These responses are transmitted to mobile devices via Bluetooth during idle periods.

#### Mechanical Characterization of the eMPatch

Mechanical stability was evaluated upon the skin penetration capacity of the MN sensor and the flexibility tests of the MN patch. To mimic the minimally invasive skin penetration manner, the process of a single MN inserting into an agarose hydrogel (1.4% agarose in PBS) was photographed by a biological inverted microscope. Mechanical compression tests were performed using a universal testing machine (HY-0580, Hengyi). The initial distance between the MN tips and the workbench was set to 1.0 mm. The compression rate and force threshold were set to 0.5 mm min^−1^ and 10  N. The load and displacement were recorded every 0.1 s to plot the load–displacement curve.

The stretchability of interconnects was first evaluated by comparing the resistance of the stretchable silver ink composed of different ratios of SIS under different strains (3706A, Keithley). The electrochemical responses of each MN sensor under different mechanical deformations (i.e., bending and twisting) in the aISF with standard analytes were measured by chronoamperometry and OPC test using an electrochemical workstation (1040c, CH Instruments). To characterize the after-bending stability, electrochemical measurements were performed before and after 100, 200, 300, 400, and 500 cycles of mechanical bending of the same MN patch. The sensing stability of SIS-based and PI-based MN patches under twisting deformation was demonstrated by comparing the motion artifacts generated during chronoamperometric measurements of MN sensors in a 5 mM [Fe(CN)_6_]^3−/4−^ solution. Sequential mechanical vibration was applied to the sensing area for 60 s. The PI-based MN patch was fabricated with the same configuration using PI substrates.

#### Finite Element Simulation for the eMPatch

The stretchability of the MN patch was evaluated by finite element simulations (COMSOL Multiphysics software, version 6.2). Two models were developed by applying a 15% stretching deformation on a flexible MN patch with or without a PET film. It was assumed that the rigid MN sensors were supported by the PET film, and therefore, the electrochemical measurements remained stable, and no detachment of MN was observed under stretching deformation. Three types of materials were introduced to the models, including a flexible elastomer (SIS) with elastic modulus *E* = 1 MPa, Poisson’s ratio *ν* = 0.5, a non-stretchable PET film with elastic modulus *E* = 7 GPa, Poisson’s ratio *ν* = 0.32, and a rigid PSMN with elastic modulus *E* = 3.5 GPa, Poisson’s ratio *ν* = 0.35. The boundary conditions are horizontal stretching deformation and limited displacement in the vertical direction. The stress-stretch relationship can be obtained through Hooke’s law:

1 $$\varepsilon = \frac{\sigma }{E}$$

2 $$\gamma =\frac{\tau }{G}$$

where *ε* was the strain, *σ* was the stress*, γ* was the shear strain, *τ* was the shear stress, and *G* was the shear modulus. The relationship between G and E was:

3 $$G = \frac{E}{2(1 + \nu )}$$

#### In Vivo Evaluation for Continuous Monitoring

In vivo evaluation and validation of the eMPatch were carried out in compliance with the guidelines and ethical regulations of protocol R20230603, which was approved by the Animal Ethics Committee of East China Normal University. Continuous multiplexed monitoring was conducted on male SD rats (6–8 weeks, 180–200 g), which were obtained from SPF Biotechnology and accommodated in a standard laboratory animal center (12-h light/dark cycle, 50 ± 10% relative humidity, and a temperature of 23 ± 2 °C) for one week before all experiments. SD rats (n = 15) were categorized into a normal group (NORM), a high glucose group (HG), and a high UA group (HUA). After overnight fasting for 12 h, the rats were induced to anesthesia with isoflurane (5% mg kg^−1^), and the dorsal hair was shaved off, followed by disinfection with 75% ethanol for two times. The HG group was intraperitoneally injected with 10% glucose at a dose of 0.05 mL 10 g^−1^, and the HUA group was treated with 25 mg mL^−1^ PO and HX (dispersed in 0.5% CMC) at 0.05 mL/10 g, while the NORM group received saline as a control. The eMPatch was applied to the bare skin, leaving pin electrodes exposed for the connections of the wireless electronic system. Chronoamperometric and OPC measurements were run for 5 min to stabilize the signals. Parameters were used the same as in vitro demonstrations. A commercial glucometer (UG-12, Sinocare) was used for glucose validation. Tail blood was collected for the validation of UA, cholesterol, sodium, and potassium using commercial colorimetric assay kits. The values of pH were measured by a commercial micro pH meter (9826BN, Orion).

#### In Vivo Evaluation for Long-Term Monitoring

For long-term monitoring, eight-week-old male SD rats (n = 9) were purchased from SPF Biotechnology and divided into three groups randomly with three rats each: a control group (CON) fed with a standard diet; a high-fat-high-fructose diet (HFFD) group fed with a DIO diet; a high-fat-high-fructose-high-salt (HFFSD) group fed with a DIO diet mixed with 8% (w/w) NaCl. Long-term monitoring was carried out by measuring the biochemical signals using the eMPatch once a week for 4 weeks. Five-minute measurements were allowed for the signal stabilization, and the following data from the biosensors were collected for the next 5 min. The body weight and gold-standard measurements of the rats were recorded every week.

#### Algorithm Development Platform

To ensure the objectivity and reliability of the model’s evaluation, all the algorithms are implemented in the interactive programming environment Jupyter Notebook with Python 3.8.10. All the programs are carried out on the Windows 11 (× 64) operating system, powered by an Intel (R) Core (TM) i7-14650HX CPU and NVIDIA GeForce RTX 4060 Laptop GPU. To accelerate the training process of deep learning, a GPU is used for parallel computing. CUDA™ (Compute Unified Device Architecture) is a general-purpose parallel computing architecture launched by NVIDIA, enabling GPUs to solve complex computing problems. It includes the CUDA instruction set architecture (ISA) and the parallel computing engine inside the GPU. In this study, we utilized the CUDA-enabled version of PyTorch (V1.12.1) and scikit-learn (V1.3.2) to optimize computation.

#### Data Preprocessing

Data preprocessing is a critical step to ensure the quality of input data for machine learning models. For raw data, data standardization must be performed before feeding it into the model. The Z-score standardization technique is employed, scaling feature values to a standardized form with a mean of 0 and a standard deviation of 1. This ensures that different feature values have a balanced influence on weight updates during model training, enhances the model’s stability, and accelerates the convergence process. For the standardized dataset, a fivefold cross-validation method is applied to split the data into training and validation sets. This approach ensures effective data utilization while mitigating the risk of overfitting. The entire dataset is evenly divided into five equally sized subsets. The model is trained and validated five times, with each iteration using a different subset as the validation set and the remaining four subsets as the training set. This means that each subset serves as the validation set once and is used as part of the training set in the other four iterations, ensuring the model’s performance remains balanced across all data. During the subset partitioning process, it is essential to maintain balanced class distributions for classification tasks and target value distributions for regression tasks. This helps to reduce bias caused by imbalanced data distributions and ensures the accuracy and fairness of the model evaluation. The metrics used in ML evaluations are provided in the Supporting Information.

## Results and Discussion

### Design of the eMPatch

The wearable eMPatch consists of a modular MN sensing component functionalized with different bio-recognition elements for molecular analyses and a flexible polymer substrate with mechanical elasticity and robustness for intimate skin conformability. The MN sensing component was designed with three enzyme-based sensors, three ion-selective sensors, a shared counter electrode (CE), and a shared reference electrode (RE), which was assembled separately on the laser-patterned SIS elastomer substrate as an integrated biosensor system. The stretchable interconnects were attached to the MN patch by screen printing (Fig. S1). Then, the sandwich-structured eMPatch was fabricated by attaching a patterned dressing film and a PET backing to the top and bottom of the patch using commercial medical tapes, respectively (see Experimental Section and Figs. S2 and S3 for details of the fabrication process). Biochemical signals were collected from the multilayered functionalized MN sensors by chronoamperometry and open circuit potential (OCP) measurements for multiplexed monitoring. Due to the high similarity between the composition of blood and ISF, glucose, UA, cholesterol, Na^+^, K^+^, and pH were targeted for their crucial roles in metabolic pathways. A self-developed electronic system comprising a microcontroller unit, a potentiostat, and a Bluetooth module was interfaced for multichannel signal collection, processing, and wireless communication. Further integrated with an ML pipeline, the eMPatch can be customized to pinpoint the physiological status with interpretative and predictive outputs, thereby offering a dynamic perspective for home-care management of health and chronic diseases with holistic monitoring and clinical analysis.

### Fabrication and Characterization of the MN Sensor

**Fig. 2:**
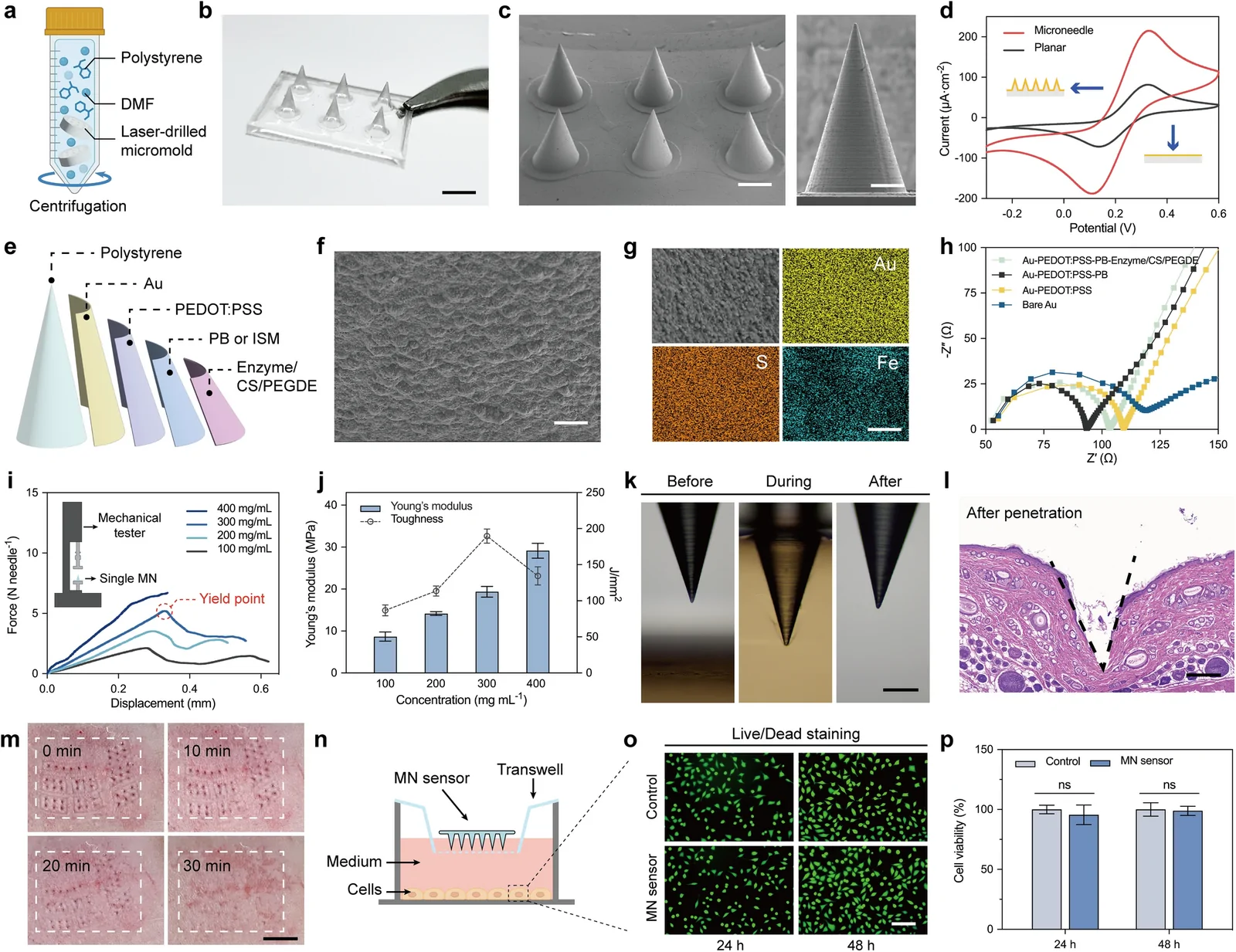
Design and characterization of the MN sensor. **a** Schematic showing the fabrication of the MN. **b** Photograph of the MN. Scale bar, 1 mm. **c** SEM images of the MN (left) (scale bar, 400 μm) and one needle (right) (scale bar, 100 μm). **d** CV scans of the Au-sputtered MN and planar electrodes with similar electrode areas in a 5 mM [Fe(CN)_6_]^3−/4−^ solution. Scan rate, 50 mV s^−1^. **e** Schematic showing the multilayer modifications of the MN sensor. **f** SEM image of the electrodeposited PEDOT:PSS. Scale bar, 10 μm. **g** SEM and EDS mapping images of the modified MN sensor. Scale bar, 25 μm. **h** EIS measurements of the MN sensor in a 5 mM [Fe(CN)_6_]^3−/4−^ solution after each modification step: Au, PEDOT:PSS, PB, and enzyme/CS/PEGDE network. **i** Mechanical compression test of MN fabricated with different concentrations of PS. **j** Relationship between Young’s modulus and toughness. Error bar indicates SD from three replicates (n = 3). **k** Microscopic images of an MN before/during/after penetrating artificial skin tissue. Scale bar, 200 μm. **l** H&E staining image of the rat skin tissue after MN insertion. Dash lines indicate the micropore formed by the insertion manner. Scale bar, 100 μm. **m** Zoom-in images showing the recovery process of 0, 10, 20, and 30 min after MN insertion. Scale bar, 0.5 cm. **n** Schematic showing the cell viability experimental setup. **o** Fluorescent micrographs exhibiting the viability of mouse fibroblast L929 after 24 and 48 h of incubation. Scale bar, 100 μm. Error bar indicates SD from three replicates (n = 3). **p** Cell viability results of mouse fibroblast L929 after incubating with/without the MN sensors for 24 and 48 h. ns, *P* > 0.05. One-way ANOVA with Dunnett’s test

The polystyrene-based MN (PSMN) was fabricated using the conventional demolding technique based on a previously reported study [33]. Briefly, a PS solution was prepared in DMF and cast into a laser-drilled poly(dimethylsiloxane) (PDMS) micromold by centrifugation (Fig. 2a). After 12 h overnight drying, an array of conical PSMN with six independent MN was demolded for further modification (Fig. 2b). The as-fabricated MN array was arranged with 1000 μm in height, 600 μm in base diameter, and 800 μm in needle-to-needle space, which ensured the balance among surface area, mechanical strength, and insertion efficiency (Fig. S4). Scanning electron microscopy (SEM) images showed the zoom-in morphology of a sharp and intact needle tip with a radius of 10 μm for reliable skin penetration (Figs. 2c and S5). The 3D architecture of MN promoted mass transport and provided a large geometric surface (exposed surface area of the MN cone) for analyte coupling compared to the planar electrode [34]. In principle, a higher aspect ratio of MN, as well as a smaller needle tip radius, can further elevate the electrochemical response [35]. Cyclic voltammetry (CV) scans of the Cr/Au sputtered MN (AuMN) and the planar electrode with a similar electrode area demonstrated that the redox peak of the MN electrode was 2.8 times more prominent than that of the planar electrode (Fig. 2d). To render the multiplexed sensing in ISF, a versatile layer-by-layer modification strategy was proposed for the MN sensor (Fig. 2e). The solid MN were first sputtered with a thin Cr/Au layer to form conductive substrates. A poly(2,3-dihydrothieno-1,4-dioxin):poly(styrenesulfonate) (PEDOT:PSS) film was electropolymerized onto the Au-coated MN with an optimized EDOT concentration of 0.2 wt%, enabling a stable solid-contact layer for the detection of ion species (Na⁺, K⁺, and H^+^) (Fig. S6). For enzymatic sensing, the Au-PEDOT:PSS-modified MN were further functionalized with a Prussian Blue (PB) mediator layer. Enzymatic cocktails were subsequently immobilized onto the MN surface using a chitosan-poly(ethylene glycol) diglycidyl ether (PEGDE) crosslinked network. Notably, the final tip diameter of the fully functionalized MN electrodes remained below 50 μm, enabling effective skin penetration without compromising mechanical integrity or insertion capability (Figs. S7 and S8) [36].

The rough nature of PEDOT:PSS with a high electrochemically active surface area (ECSA) provides sufficient geometry for the loadings of mediators, enzymes, and crosslinking agents (Fig. 2f) [37]. The ECSA was estimated from the experimentally obtained double-layer capacitance, demonstrating the higher ECSA of the PEDOT:PSS-coated AuMN than that of planar Au and bare AuMN (Figs. S9 and S10). Energy dispersive X-ray spectroscopy (EDS) analyses further confirmed the successful modification of PEDOT:PSS and PB by the presence of sulfur (S), iron (Fe), and potassium (K) (Figs. 2g and S1!). The thickness of PB was tailored with different scanning cycles based on the levels of biomarkers in ISF (Fig. S12). In principle, a thicker PB layer leads to a wider linear range while the thinner exhibits higher sensitivity toward low-concentration biomarkers due to the faster charge transfer kinetics [38]. Therefore, the as-modified MN electrode demonstrated a diffusion-controlled mode for mass transport with superior conductivity, enabling efficient electrochemical performance for multiplexed sensing (Fig. S13). The pH sensor was prepared by the electropolymerization of hydrogen ion-sensitive polyaniline (PANI) on the PEDOT:PSS decorated AuMN (Fig. S14). The modification steps were characterized by EIS and CV scans (Figs. 2h and S15). The PEDOT:PSS decoration led to an increased peak current in the CV plot and lower impedance in the Nyquist plot, indicating the film could promote both electronic and ionic charge transport for preparing enzymatic and ion-selective sensors [39].

The skin penetration capability of the MN was comprehensively evaluated through the following experiments. The mechanical strength of the MN synthesized with different concentrations of PS (100, 200, 300, and 400 mg mL^−1^) was investigated through a compression test (Fig. 2i). The MN with 100 mg mL^−1^ could withstand a maximum force of 2.1 N (0.35 N needle^−1^) without obvious fracture, which was greater than the required robustness for skin penetration [40]. However, the 400 mg mL^−1^ PS with a higher yield point (5.8 N) was subject to poor toughness, which may lead to brittle fracture of needle tips after MN insertion or during physical movement (a sharp drop of force indicated the yield point of needle tips). Therefore, an optimized concentration of 300 mg mL^−1^ was selected for further tests according to the correlation between Young’s modulus and toughness (Fig. 2j). An agarose-prepared artificial skin tissue was used to mimic the skin penetration process, where an intact needle tip was observed before and after the MN insertion (Fig. 2k). In Parafilm (in vitro) and porcine skin (ex vivo) tests, the MN successfully penetrated Parafilm and porcine skin with well-defined microholes, confirming effective penetration and structural integrity of the MN (Fig. S16). As demonstrated in the Hematoxylin and eosin (H&E) images, the MN accurately reached the ISF in the dermis layer without damaging subcutaneous capillaries (Fig. 2l). The recovery capability was assessed by monitoring the MN-induced trace over time (0, 10, 20, and 30 min) (Fig. 2m). A group of noticeable microholes on the rat’s dorsal skin was observed after the removal of the MN (0 min), which recovered within 30 min without leaving any visible skin damage or irritation. The biocompatibility of the MN sensor was evaluated by the cell viability assay and Live/Dead staining (Fig. 2n). Compared with the control group, no significant effect on cell viability was observed during the 24 and 48 h incubation period, indicating the excellent biocompatibility of the MN sensor for long-term monitoring (Fig. 2o, p). In addition, photographs of rat skin after prolonged wear of the MN for 7 days showed no visible signs of erythema or inflammation (Fig. S17). H&E staining of major organs (heart, liver, spleen, lung, and kidney) collected at 1 day and 7 days post-wearing revealed no noticeable pathological alterations compared with the control group, indicating strong biocompatibility of the eMPatch during prolonged use (Fig. S18).

### Electrochemical Performance of the Integrated eMPatch

**Fig. 3:**
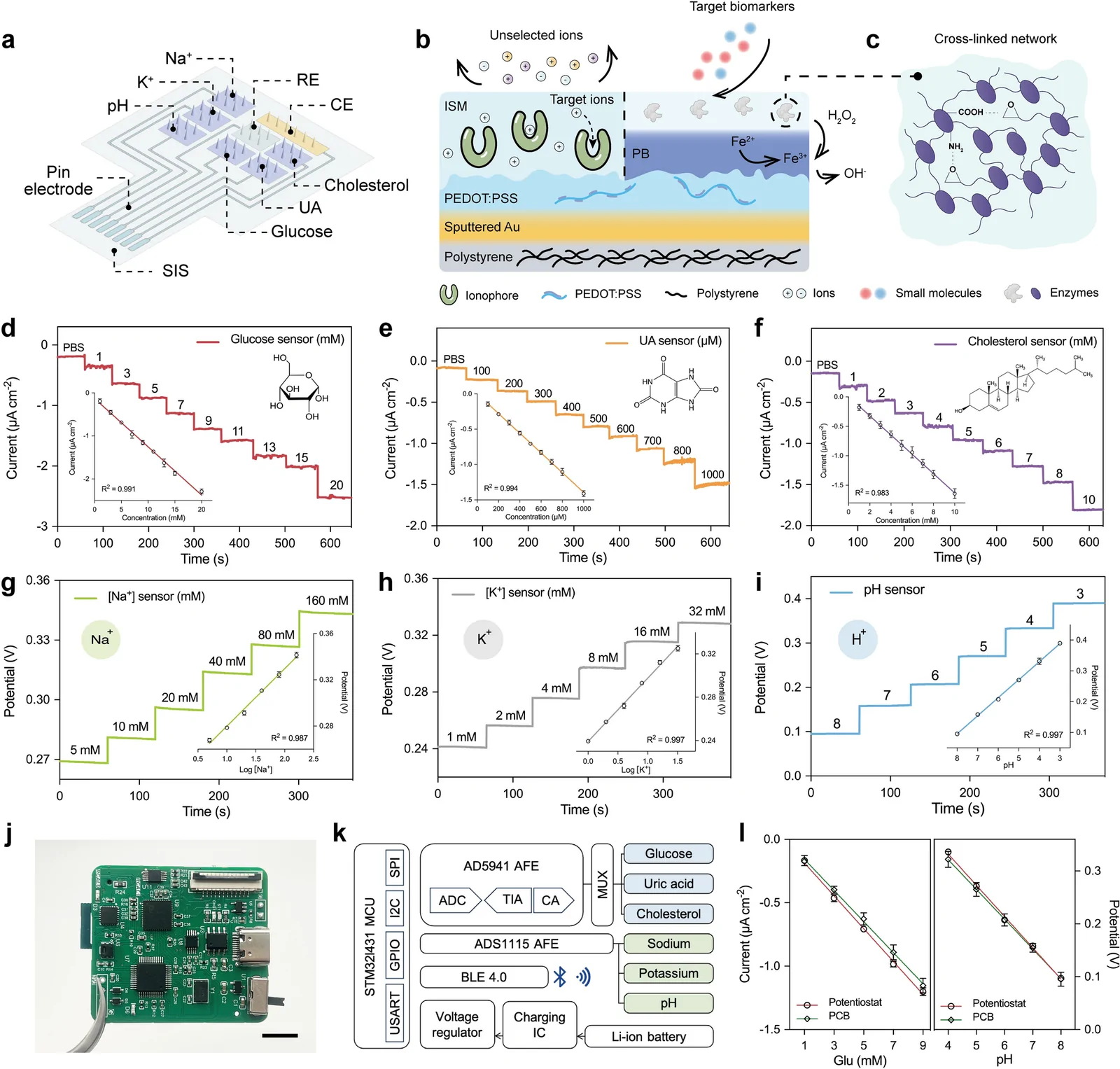
Electrochemical characterization of the eMPatch. **a** Schematic of the eMPatch that comprises enzymatic and ion-selective sensors for glucose, UA, cholesterol, Na^+^, K^+^, and pH monitoring. **b-c** Schematic demonstrating the mechanism of the MN sensors for enzymatic and ion-selective sensing (**b**) and the cross-linked enzyme/CS/PEGDE network (**c**). **d–f** Chronoamperometric responses of the glucose (**d**), UA (**e**), and cholesterol sensors (**f**) to target analytes. Insets, the corresponding calibration curves in different ranges. Error bar indicates SD from three replicates (n = 3). **g–i** OCP responses of the Na^+^ (**g**), K^+^ (**h**), and pH sensors (**i**) to target analytes. Insets, the corresponding calibration curves in different ranges. Error bar indicates SD from three replicates (n = 3). **j-k** Photograph (**j**) and schematic diagram (**k**) of the self-developed electronic system for wireless monitoring. Scale bar, 1 cm. **l** Comparison of the linear fitted curves of glucose and pH measured by the electronic system and electrochemical workstation (CHI 1040c). Error bar indicates SD from three replicates (n = 3)

The eMPatch consists of amperometric glucose, UA, cholesterol sensors, and potentiometric ion-selective Na^+^, K^+^, pH sensors (Fig. 3a), enabling the enzymatic sensing based on the PB mediator with a low redox potential, and ion-selective sensing with PEDOT:PSS as the intermediate solid-contact layer for the efficient ion-to-electron transduction (Fig. 3b, c). The feasibility of each sensor was evaluated in standard solutions containing corresponding analytes with physiologically relevant concentrations.

The electrochemical performance of enzymatic sensors was displayed with representative chronoamperometric responses to glucose (0–20 mM), UA (0–1000 μM), and cholesterol (0–10 mM) (Fig. 3d–f??). Strong linear correlations of glucose, UA, and cholesterol sensor (*r* = 0.991, 0.994, 0.983) were obtained with the sensitivities of 0.1122 μA cm^−2^ mM^−1^, 0.001395 μA cm^−2^ μM^−1^, and 0.1637 μA cm^−2^ mM^−1^, respectively. The limit of detection (LOD) was calculated as 0.39 mM, 2.7 μM, and 0.52 mM, respectively (LOD = 3.3*σ*/*S*, where *σ* is the standard deviation of the baseline noise and *S* is the slope of the calibration curves). The results indicated the real-world practicability of the eMPatch for glucose, UA, and cholesterol monitoring over the physiologically relevant ranges of 4.4–6.6 mM, 130–460 μM, and 2.9–5.2 mM [41–43]. For the ion-selective sensors, well-defined OCP curves were obtained with near-Nernstian sensitivities of 52.87 mV (*r* = 0.987) and 58.46 mV (*r* = 0.997) per decade concentration, covering the physiological ranges of Na^+^ (135–145 mM) and K^+^ (3.5–5 mM) (Fig. 3g, h??) [44]. The pH sensor exhibited a linear response of 57.61 mV pH^−1^ (*r* = 0.997) by switching the protonation status of the PANI surface over a physiologically relevant range of 6.6 to 7.6, which is wider than the values in the blood due to the lack of buffer molecules in ISF (Fig. 3i??) [45]. The accuracy of the calibrated eMPatch was validated against the commercial colorimetric assay kits in the aISF (Fig. S19). In addition, the influence of pH and temperature on the eMPatch was optimized under different environmental conditions (Fig. S20) [46].

The eMPatch displayed high sensitivity against potential interfering species commonly found in ISF (Fig. S21). All the MN sensors showed negligible signal drifts within 6% to each addition of other interference analytes, followed by notable increases upon incremental addition of the target analytes. The repeatability of eMPatch patches toward cyclically elevated concentrations was exhibited, which indicated strong reliability against drastic level changes during continuous monitoring (Figs. S22 and S23). To further evaluate the stability of the eMPatch over long-term monitoring, all MN sensors were continuously operated in the aISF with corresponding analytes in physiologically relevant concentrations for 120 min, during which stable electrochemical signals with more than 88% signal retention were maintained for targeted biomarkers (Fig. S24). In addition, the eMPatch maintained a steady-state response with a relative standard deviation (RSD) below 2.64% for all MN sensors during a 14-day storage experiment, demonstrating the reliable shelf-life stability (Fig. S25). The eMPatch exhibited high reproducibility with RSD within 2.21% by comparing the normalized outputs from five batches (eight patches for each batch, n = 8), demonstrating good stability of the as-proposed eMPatch for mass production and universal application (Fig. S26).

The fully integrated eMPatch was also interfaced with a self-developed electronic system for multiplexed signal processing and wireless communication. The schematic illustration, optical image, and overview of the system are shown in Fig. 3j, k???, which was designed with an electrochemical analog front end (AFE) providing the circuitry for multiplexed chronoamperometric measurements and signal conditioning (amplification and filtering), and an external analog-to-digital (ADC) unit incorporated for multichannel potentiometry. Data acquisition, command processing, and task scheduling were managed by a low-power microcontroller unit (MCU). A low-power Bluetooth Low Energy (BLE) module was utilized for wireless communication. Power of the electronics was sourced by a lithium-ion battery with a USB Type-C compatible charging circuit for up to six hours of continuous operation. The detailed circuit, firmware, and software designs are shown in Figs. S27 and S28. The accuracy of the AFE sensing module and the ADC chip was strongly validated through the well-established calibration curves between the wireless electronic system and the electrochemical station (glucose and pH measurements as examples) (Fig. 3l).

### Mechanical Evaluation of the eMPatch

**Fig. 4:**
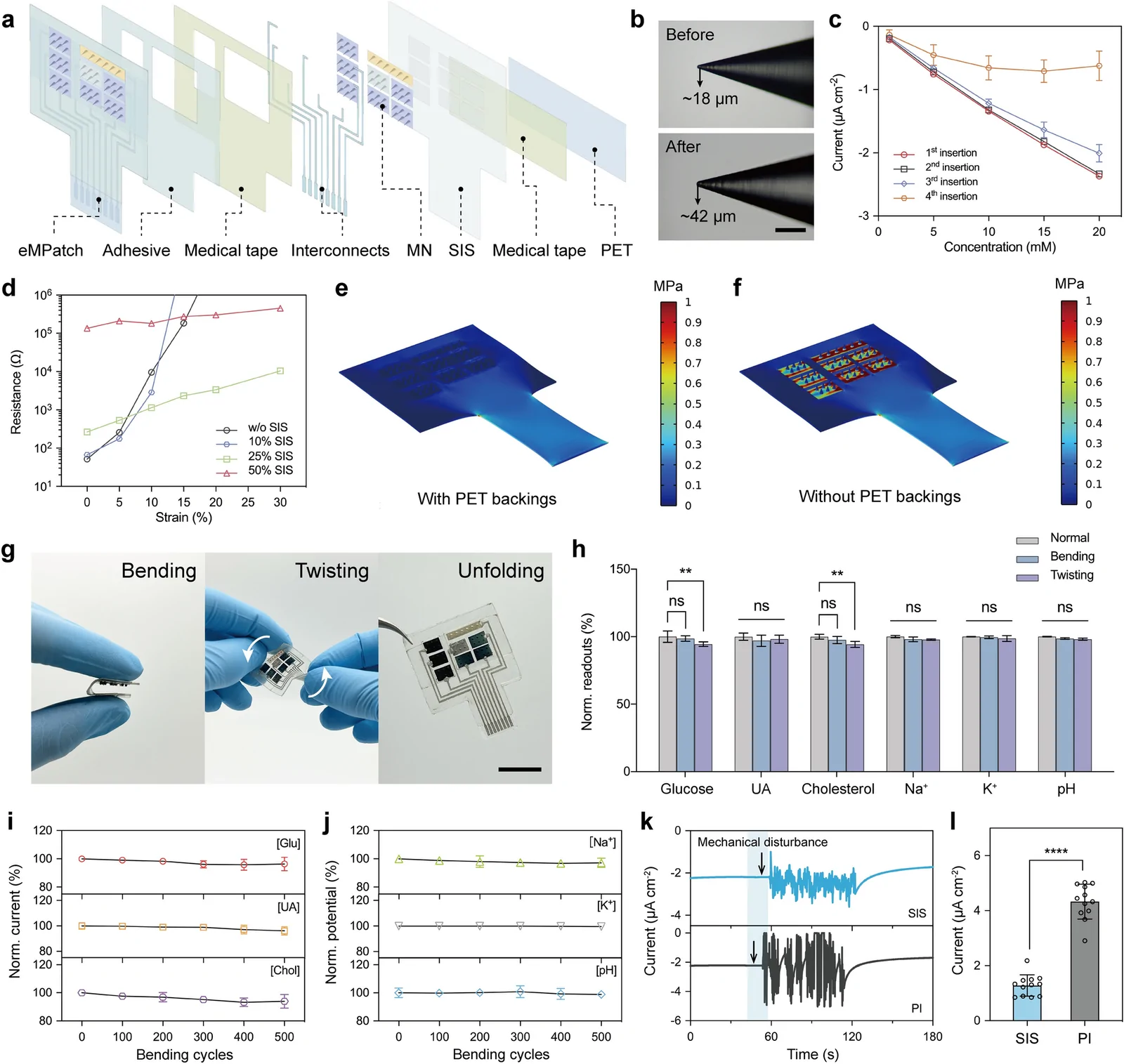
Mechanical characterization of the eMPatch. **a** Exploded view detailing the layer-by-layer configuration of the eMPatch. **b** Images showing the MN tips before and after multiple insertions into rat skin. Scale bar, 200 μm. **c** The influence of different insertion times on the electrochemical responses to 5 mM glucose. Error bar indicates SD from three replicates (n = 3). **d** Resistive change of the stretchable interconnects with different ratios of SIS under varying uniaxial strain percentages. **e–f** Finite element simulations of the distribution of stress on the eMPatch with (**e**) and without (**f**) the PET backings under a horizontal strain percentage of 15%. **g** Optical images showing the mechanical resilience of the eMPatch under bending (left) and twisting (middle), and after these deformations (right). Scale bar, 1 cm. **h** Normalized electrochemical readouts of each sensor to standard analyte solutions before and under mechanical deformations (Glucose: 5 mM, UA: 300 μM, cholesterol: 5 mM, Na^+^: 140 mM, K^+^: 5 mM, pH: 7.0). Error bar indicates SD from three replicates (n = 3). **P < 0.01, ns, *P* > 0.05. One-way ANOVA with Dunnett’s test. **i** Normalized current of enzymatic sensors to standard analyte solutions after every 100 stretching (vertical) cycles up to 500 cycles (Glucose: 5 mM, UA: 300 μM, cholesterol: 5 mM). Error bar indicates SD from three replicates (n = 3). Glu: glucose, Chol: Cholesterol. **j** Normalized potential of ion-selective sensors to standard analyte solutions after every 100 stretching (vertical) cycles up to 500 cycles (Na^+^: 140 mM, K^+^: 5 mM, pH: 7.0). Error bar indicates SD from three replicates (n = 3). **k** Chronoamperometric measurements of SIS-based and PI-based MN patches in a 5 mM [Fe(CN)_6_]^3−/4−^ probe with the same magnitude of mechanical disturbance simultaneously. **l** Current amplitude of motion artifacts on SIS-based and PI-based MN sensors. Error bar indicates SD from twelve measurements (n = 12). ****P < 0.0001. Unpaired, two-tailed *t*-test

The combination of rigid MN sensors and a soft skin-worn substrate allows for efficient MN sensing with excellent adaptability and conformability (Fig. 4a). Therefore, the mechanical stability of the as-proposed eMPatch was investigated when unexpected deformations occurred.

The effect of skin penetration on the eMPatch was evaluated. First, no noticeable fracture or blunting of the MN tips, as well as delamination or cracking of the MN surface, were observed after MN insertion, confirming the mechanical robustness and pierceability of the MN (Figs. 4b, S29, and S30). The variations in sensitivities and mass of the eMPatch suggested a maximum of three times insertion with acceptable integrity of sensing layers for reliable monitoring, which provided opportunities against incomplete MN penetration and MN detachment in daily activities (Figs. 4c and S31). The conductive performance of the stretchable interconnects was tailored by the concentration of SIS. A weight ratio of 25% SIS exhibited both stable resistance and stretchability under up to 30% strain deformation (Fig. 4d). The thin PET backings underneath the eMPatch ensured the structural integrity by providing supportive strength against undesirable deformations during on-body operations. Finite element simulations demonstrated the uniform distribution of stress on the PET-protected eMPatch compared to the counterpart without PET backings, which exhibited higher principal stress on the MN sensors under 15% strain (Fig. 4e, f). The results indicated a higher risk of MN delamination of the eMPatch without PET backings by stress-induced deformations (Fig. S32). The excellent mechanical resilience of the eMPatch after different deformations is shown in Fig. 4g. The patch was unfolded with no apparent device disassembly after bending for 180° and neck twisting for 90°. The influence of these deformations on the sensing performance was also evaluated by an ex vivo experimental setup (Fig. S33). As shown in Fig. 4h, bending exerted negligible effects during the electrochemical measurements of all MN sensors. Although the effects of twisting indicated slight signal deviations of 5.7% and 4.1% for glucose and cholesterol sensors, the concentration-correlated readouts were still maintained within the physiological range. Reliable mechanical stability against the bending manner was observed by recording the electrochemical signals of each MN sensor after every 100 repetitive bending cycles in the aISF (Fig. 4i, j). Twisting-induced motion artifact noises were quantified between an SIS-based and a non-stretchable polyimide (PI)-based MN patch, showing four times lower vibration amplitude generated from the SIS-based eMPatch compared with the PI-based patch (Fig. 4k, l).

### In Vivo Validation of the eMPatch for Multiplexed Monitoring

**Fig. 5:**
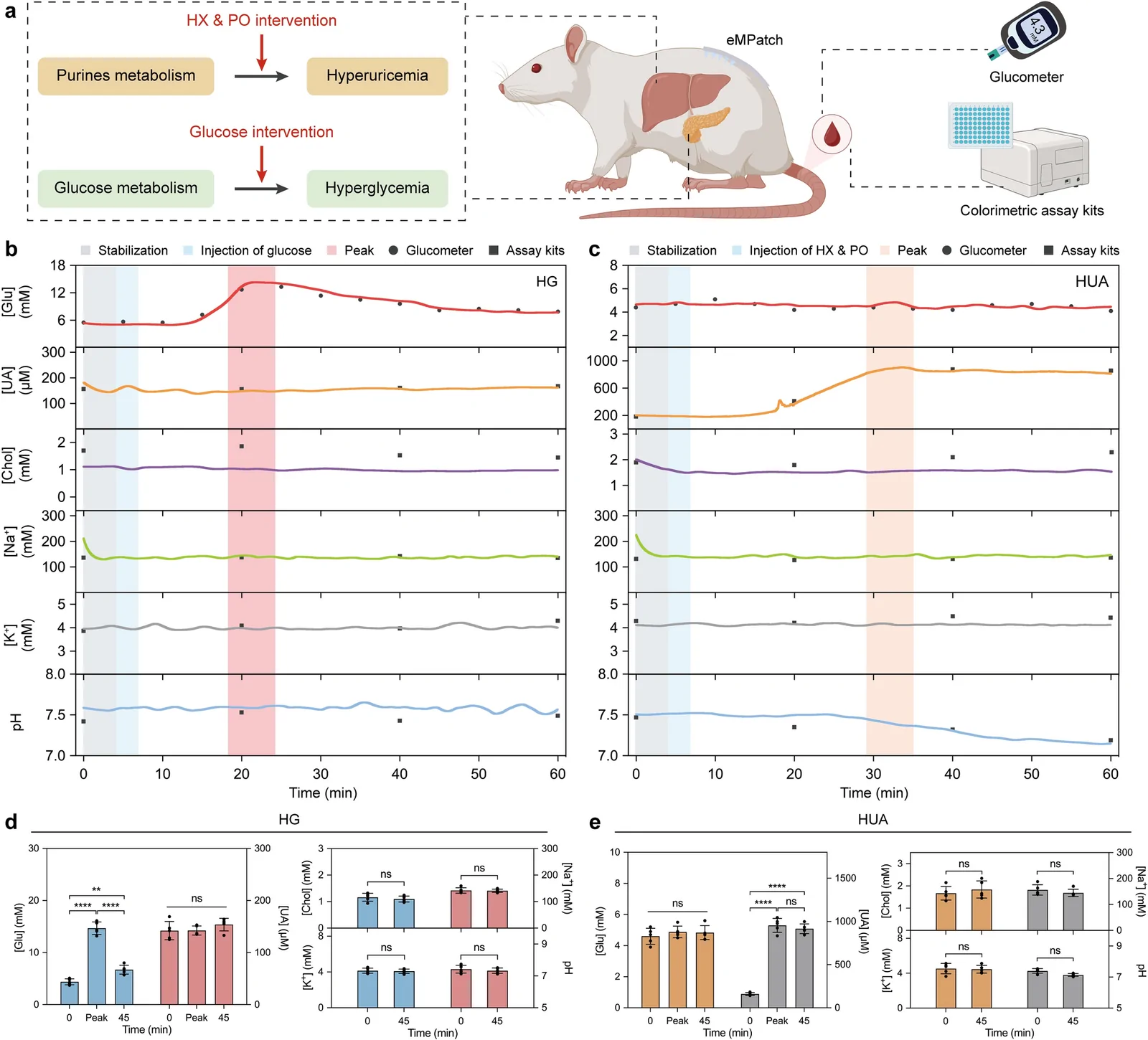
In vivo evaluation of the eMPatch on rat models. **a** Schematic demonstrating the animal experiment process. HX, hypoxanthine; PO, potassium oxonate. **b**, **c** Continuous monitoring of multiplexed biomarkers in rat groups that received different treatments for an hour. **d**, **e** Comparison of the targeted biomarkers in ISF in the stage of: after overnight fasting, peak, and 45 min after the glucose (**d**) and HX & PO (**e**) treatments. Error bar indicates SD from five rats (n = 5). ^**^P < 0.01, ^****^P < 0.0001, ns, *P* > 0.05. One-way ANOVA with Tukey’s test. (Note: some graphics are created with Biorender.com)

To demonstrate the feasibility of the eMPatch for continuous monitoring of physiologically relevant biomarkers in ISF, controlled experiments were performed on three groups of Sprague–Dawley (SD) rat models using metabolic interventions on the levels of glucose and UA, which tend to fluctuate rapidly due to daily dietary intake and activities (Fig. 5a). Briefly, the high glucose group (HG) was intraperitoneally injected with glucose solution while the high UA group (HUA) was treated with hypoxanthine (HX) and potassium oxonate (PO), which are the typical UA precursor and uricase inhibitor. The normal group (NORM) was injected with saline as a control for the experimental comparison. In vivo evaluation of the eMPatch was performed through dynamic monitoring of physiological variations in the three groups after interventions (Fig. 5b, c). The level of each biomarker was extracted for comparison in the stages of: after overnight fasting, peak, and 45 min after intervention (Fig. S34). The performance of the eMPatch was validated in comparison with a commercial glucometer and colorimetric assay kits. The glucometer was measured every 5 min, and assay kits were tested every 20 min.

For the HG group, the glucose levels increased rapidly after the injection of glucose solution, reaching peak concentrations at ~ 20 min, followed by a ~ 30 min return to the baseline levels, indicating a well-defined insulin-regulated metabolism [46]. The trend of glucose levels in ISF and blood exhibited high similarity based on the results obtained using the standard glucometer (Fig. 5d). For the HUA group, stable dynamic signals of UA were observed for the initial ~ 15 min, followed by a substantial increase of averaged UA levels from ~ 160.2 to ~ 1015 μM, and remained consistent for ~ 30 min as the result of HX and PO administration (Fig. 5e) [47]. Compared with the experimental groups, all the dynamic profiles of the NORM group remained relatively stable responding to the intraperitoneal injection of saline (Fig. S35). Therefore, these results demonstrated the reliable capability of the eMPatch for real-time monitoring of physiologically relevant biomarkers during daily activities.

### Evaluation of the eMPatch for Deep Learning-driven Personalized Health Management

To evaluate the performance of the eMPatch for long-term health evaluation, in vivo experiments using diet-induced rat models were conducted: a control (CON) group, a high-fat-high-fructose diet (HFFD) group, and a high-fat-high-fructose-high-salt diet (HFFSD) group (Fig. S36). A biomarker dataset was constructed based on all sensor responses collected from the three groups (Figs. S37 and S38, Table S2). A pattern recognition algorithm was applied to this dataset to achieve high-performance real-time analysis of rat health.

**Fig. 6:**
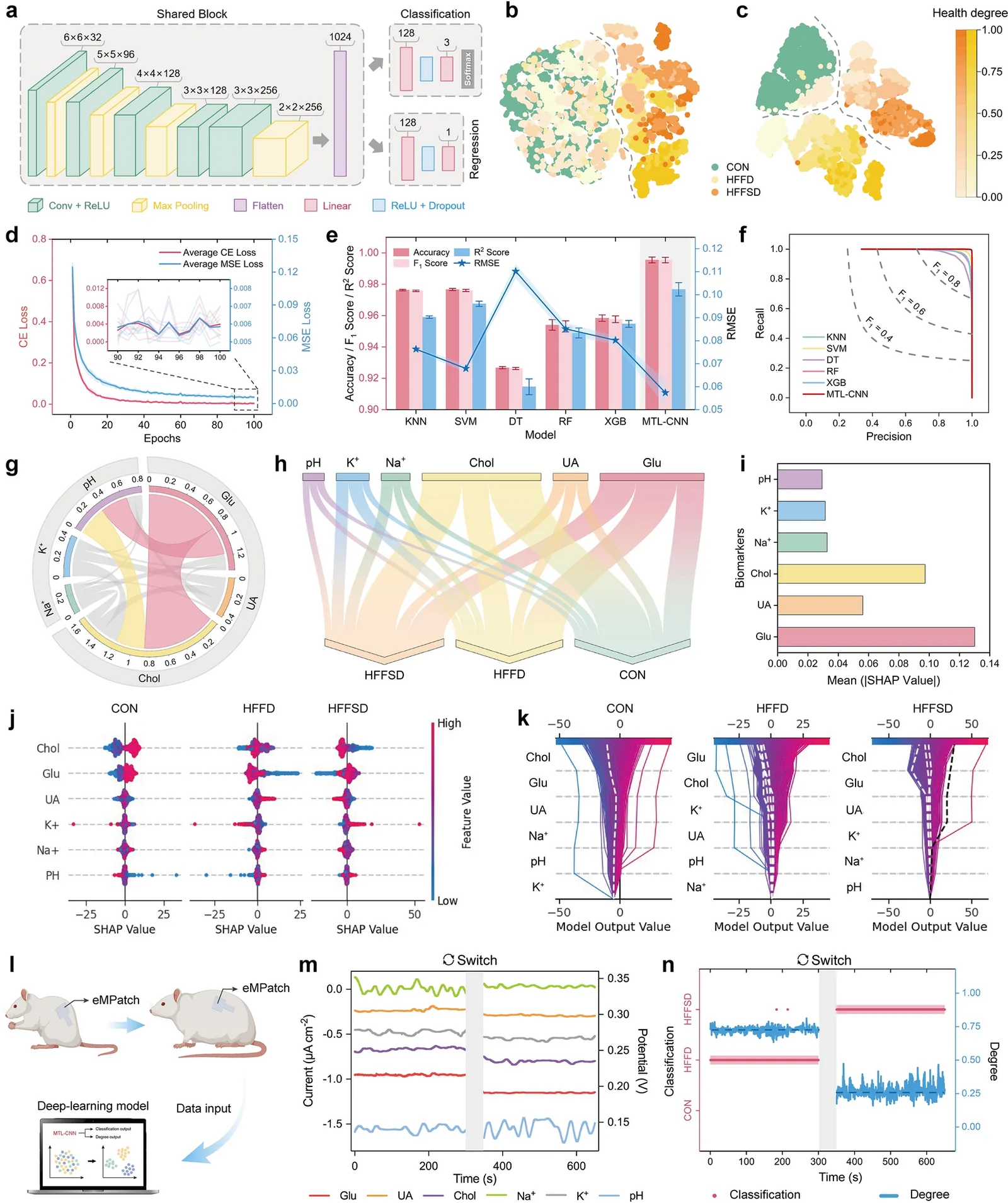
Deep learning-driven health evaluation. **a** Schematic of the structure of the MTL-CNN model. **b** t-SNE clustering analysis of the original biomarker dataset, visually demonstrating feature distribution in a two-dimensional space. **c** t-SNE clustering analysis of deeply embedded features, illustrating the automatic feature extraction capability of the MTL-CNN. **d** Training curves of cross-entropy loss and MSE loss under fivefold cross-validation. **e** Validation performance of classical ML algorithms and deep learning algorithms for health evaluation. KNN, K-Nearest Neighbors; SVM, Support Vector Machine; DT, Decision Tree; RF, Random Forest; XGBoost, Extreme Gradient Boosting. **f** Precision-recall curves of different ML models used for health assessment. **g** Chord graph showing the relative correlations of data corresponding to different MN sensors. **h** Sankey diagram based on SHAP analysis, indicating the relative contributions of different biomarkers, used as model input features for health classification. **i** SHAP analysis of the health degrees evaluation process, highlighting the importance of different features in the regression task. **j** SHAP summary plot for health classification based on 1000 instances, with each axis displaying the distribution of SHAP values for validation samples corresponding to the respective feature. **k** SHAP decision plot explaining the process by which the MTL-CNN model utilized molecular features to classify health conditions. **l** Schematic showing the deep learning-coupled eMPatch for health management. **m** Real-time monitoring of physiologically relevant biomarkers from randomly selected rat models. **n** Output of the MTL-CNN model based on the sensor data acquired by the eMPatch

In this work, a multi-task convolutional neural network (MTL-CNN) model was designed to simultaneously perform health conditions classification and health degrees evaluation. The deep learning model consists of three core modules, including a hard-shared block and two task-specific branches (Fig. 6a, Table S3). The shared block, consisting of five convolutional layers, is shared by both tasks and automatically extracts features from the raw electrochemical data input into the model. The extracted deep-level features are then fed into two branches, one dedicated to classification and the other to regression. Feature extraction was validated by the t-distributed stochastic neighbor embedding (t-SNE) dimensionality reduction and clustering, where overlapping and disordered data distribution was observed by projecting high-dimensional raw data into two-dimensional space (Fig. 6b). However, the embedded high-level features extracted by the MTL-CNN exhibited more distinctive clusters, demonstrating the superior automatic feature extraction capability of the deep learning model (Fig. 6c).

The MTL-CNN model was trained and validated using fivefold cross-validation. The loss values for both tasks significantly decreased while the evaluation metrics closely overlap and steadily increase across the five folds, indicating the model’s highly improved fitting performance on the training set (Figs. 6d and S39). The model strongly outperformed classical ML models, with the average classification accuracy of 0.996 for health conditions and the average R^2^ score of 0.977 for health degrees evaluation, demonstrating the high accuracy, strong robustness, and excellent inter-task synergy of the MTL-CNN in health management (Fig. 6e). The precision-recall (PR) curve of the MTL-CNN model achieved a strong balance, indicating robust predictions of physiological conditions without misclassifications (Fig. 6f). In contrast, the classical models exhibited higher prediction errors owing to the inadaptation to complex patterns or overfitting (Fig. S40). The high accuracy of the confusion matrix and the strong fit of the violin plot further validated the advantages of the MTL-CNN model in multi-task scenarios (Figs. S41 and S42).

The Pearson correlation coefficients between the obtained data from the eMPatch were presented in Fig. 6g. The strongest association between glucose and cholesterol reflected a highly consistent relationship in metabolic pathways. The distribution of correlations provided powerful insights into targeted biomarkers in the decision-making logic of the model. To assess the feature contribution of each biomarker, SHapley Additive exPlanations (SHAP) analysis was conducted. In this regard, glucose and cholesterol emerged as major shared features with high SHAP values in both tasks, indicating their critical roles in both health classification and prediction (Fig. 6h, i). In the classification task, cholesterol made the highest contribution, primarily distinguishing between the CON and the experimental groups while glucose focused more on differentiating variations between the HFFD and HFFSD groups. However, glucose displayed significantly higher SHAP values than cholesterol for the assessment of health degrees, reflecting its direct regulatory role in metabolic pathways.

The SHAP summary plots and decision plots offered more granular insights into feature contributions. The distribution and direction of feature contributions confirmed the important role of glucose and cholesterol in classifying health conditions (Fig. 6j). Additionally, the notable but opposite direction of glucose values in the HFFD and HFFSD groups indicated that the MTL-CNN could differentiate similar yet distinct categories. The decision plots evaluated the decision-making process of the model and the misclassified samples-induced prediction bias and errors (Fig. 6k). The SHAP analyses of health degree evaluation are shown in Fig. S43. On a broader scope, the SHAP-based interpretability analyses provided deep theoretical support for the optimization of the eMPatch and enhanced the transparency and reliability of the deep learning model.

To validate the health evaluation capability of the MTL-CNN model under dynamic conditions, the eMPatch was first applied to a rat in the HFFD group for continuous monitoring of targeted biomarkers, and was then immediately transferred to a rat in the HFFSD group for further measurements. The resulting multiplexed datasets were subsequently fed into the trained MTL-CNN model (Fig. 6l). The eMPatch exhibited stable and continuous electrochemical responses throughout the measurement period (Fig. 6m). As shown in Fig. 6n, the model was able to accurately distinguish between the two health conditions and simultaneously provide a quantitative evaluation of the corresponding health degrees, which represent the continuous regression score derived from the multi-task network and reflect relative metabolic deviation from the reference health states (CON). In comparison with recent state-of-the-art wearable sensing platforms (Table S4), the eMPatch assisted with an ML algorithm highlights the competitive potential to convert ambiguous metabolic disorders into interpretative physiological indicators, providing a data-driven avenue for wearable healthcare.

## Conclusions

In this article, we proposed a smart, wearable eMPatch that enables real-time, transdermal, and multiplexed health monitoring of molecular biomarkers, which paved the way for next-generation personalized healthcare. By integrating a laser-patterned, flexible elastomer substrate, the eMPatch offered customizable sensor configurations tailored for versatile biomedical applications, while ensuring mechanical robustness for stable skin adhesion. Modular MN-based electrochemical sensors were assembled, exhibiting high selectivity and long-term stability for both enzymatic and ion-selective monitoring in dermal ISF without compromising structural integrity. Coupled with a custom-developed electronic system and deep learning-driven analytics, the eMPatch could continuously acquire high-dimensional metabolic data, enabling comprehensive physiological assessment through advanced AI-based interpretation. With the aid of the implemented MTL-CNN, the eMPatch could leverage automated feature extraction to capture complex nonlinear physiological patterns from the collected dataset. This approach enabled dual-task operations, achieving health classification with an accuracy of 0.996 and robust health degrees evaluation with an R^2^ score of 0.977. Future improvements in sensor sensitivity, energy efficiency, and fully integrated system miniaturization will be essential to advance our eMPatch toward large-scale applications. Human subject studies will be involved to further assess the translational potential for personalized health monitoring. The eMPatch will facilitate comprehensive metabolic profiling, solidifying its role as a powerful, next-generation wearable platform for precision healthcare.

## Supplementary Information

Below is the link to the electronic supplementary material.

Supplementary file1 (DOCX 13737 KB)
