# Supplementary material for: Modularly-Assembled Smart Microneedle Platform for Machine Learning-Driven Personalized Health Monitoring
Source: Nanomicro Lett. 2026 Feb 9;18:248. doi: 10.1007/s40820-026-02095-x (PMC12886675; doi:10.1007/s40820-026-02095-x)
Supplement: Supplementary file 1 — Supplementary file1 (DOCX 13737 KB) [file 40820_2026_2095_MOESM1_ESM.docx]

Supporting Information for

**Modularly-Assembled Smart Microneedle Platform for Machine Learning-Driven Personalized Health Monitoring**

Hongyi Sun^1,2^, Lechen Chen^3^, Tao Wang^4,^*, Zhuoheng Li^3^, Yi Shi^1^, Wen Lv^3^, Zhi Yang^3^, Fuzhen Xuan^4^, Min Zhang^1,^*, and Guoyue Shi^1,2^*

^1^ School of Chemistry and Molecular Engineering, East China Normal University, Shanghai 200241, P. R. China

^2^ WuHu Hospital, East China Normal University (The Second People’s Hospital of WuHu), Wuhu 241000, P. R. China

^3^ National Key Laboratory of Advanced Micro and Nano Manufacture Technology, School of Electronic Information and Electrical Engineering, Shanghai Jiao Tong University, Shanghai 200240, P. R. China

^4^ Shanghai Key Laboratory of Intelligent Sensing and Detection Technology, School of Mechanical and Power Engineering, East China University of Science and Technology, Shanghai 200237, P. R. China

*Corresponding authors. E-mail: [wangtao@ecust.edu.cn](mailto:wangtao@ecust.edu.cn) (Tao Wang); [mzhang@chem.ecnu.edu.cn](mailto:mzhang@chem.ecnu.edu.cn) (Min Zhang); [gyshi@chem.ecnu.edu.cn](mailto:gyshi@chem.ecnu.edu.cn) (Guoyue Shi)

**S1 Experimental Section**

S1.1 Classification Metrics used for ML Evaluations

The confusion matrix is a critical tool for evaluating the performance of classification models. It provides a matrix-based visualization that compares the model's predictions with the actual labels, clearly demonstrating the model's performance across different classes. The rows of the confusion matrix represent the predicted classes, while the columns represent the actual classes. The diagonal elements of the matrix indicate the number of correctly classified samples, while the off-diagonal elements represent misclassified instances (Fig. S32). For binary classification tasks, the confusion matrix consists of four fundamental elements: True Positive (TP), True Negative (TN), False Positive (FP), and False Negative (FN). TP is defined as the number of samples where the actual class is positive, and the model correctly predicts them as positive. TN refers to the number of samples where the actual class is negative, and the model correctly predicts them as negative. FP occurs when the actual class is negative, but the model incorrectly predicts it as positive. FN occurs when the actual class is positive, but the model incorrectly predicts it as negative. Using the confusion matrix, additional evaluation metrics such as accuracy, precision, recall, and F_1_ score can be derived. These metrics provide a comprehensive evaluation of the classifier's performance across different classes and help identify biases or shortcomings in the model's predictions for specific categories.

**S1.1.1 Accuracy**

Accuracy is a metric used to measure the overall correctness of predictions made by a machine learning model, particularly in classification tasks. It is defined as the ratio of the number of correctly predicted samples to the total number of samples, representing the proportion of correct predictions out of all predictions. For binary classification problems, the formula for accuracy is as follows:

$$Accuracy = \frac{TP + TN}{TP + TN + FP + FN}$$

Accuracy is the simplest and most intuitive evaluation metric for classification tasks and is well-suited for scenarios with balanced class distributions. It measures the overall correctness of a model's predictions on the entire dataset, reflecting the model's overall performance. However, when dealing with imbalanced datasets, accuracy may fail to effectively capture the model's performance on minority classes. In cases where one class dominates the dataset, the model tends to over-predict the majority class, resulting in an inflated accuracy score that overlooks the classification performance of minority classes. Consequently, the evaluation results may not comprehensively reflect the model's actual performance. Therefore, in imbalanced datasets, accuracy should typically be combined with other metrics, such as precision, recall, and F_1_ score, to provide a more comprehensive assessment of the model's performance.

**S1.1.2 Precision**

Precision refers to the proportion of samples predicted as positive by the model that are actually positive. It measures the accuracy of the model's predictions, particularly when the focus is on minimizing false positive predictions. For binary classification problems, the formula for precision is:

$$Precision = \frac{TP}{TP + FP}$$

**S1.1.3 Recall**

Recall, also known as the True Positive Rate, refers to the proportion of actual positive samples that are correctly predicted as positive by the model. It measures the model's ability to identify positive samples. For binary classification problems, the formula for recall is:

$$Recall = \frac{TP}{TP + FN}$$

**S1.1.4 F_1_ score**

F_1_ score is the harmonic mean of precision and recall, balancing the trade-off between these two metrics. It is particularly useful in scenarios where both precision and recall need to be considered simultaneously, especially when class distributions are imbalanced. The formula for the F_1_ score is:

$$F_{1} score = \frac{2 \times Precision \times Recall}{Precision + Recall}$$

**S1.1.5 Multi-class Classification Tasks**

For multi-class classification tasks, averaging techniques are employed to generalize the calculation. These techniques include micro-averaging and macro-averaging. Micro-averaging computes overall metrics by aggregating the sample counts across all classes, focusing on the model's overall performance. In contrast, macro-averaging calculates the metric for each class individually and then averages them, emphasizing equal representation for all classes. The formulas are as follows:

$${Precision}_{micro} = \frac{\sum_{i=1}^{N} {TP}_{i}}{\sum_{i=1}^{N} ({TP}_{i} + {FP}_{i})}$$

$${Precision}_{macro} = \frac{1}{N}\sum_{i=1}^{N} {Precision}_{i}$$

$${Recall}_{micro} = \frac{\sum_{i=1}^{N} {TP}_{i}}{\sum_{i=1}^{N} ({TP}_{i} + {FN}_{i})}$$

$${Recall}_{macro} = \frac{1}{N}\sum_{i=1}^{N} {Recall}_{i}$$

$$F_{1} {score}_{micro} = \frac{2 \times{Precision}_{micro} \times{Recall}_{micro}}{{Precision}_{micro} + {Recall}_{micro}}$$

$$F_{1} {score}_{macro} = \frac{1}{N}\sum_{i=1}^{N} \frac{2 \times{Precision}_{i} \times{Recall}_{i}}{{Precision}_{i} + {Recall}_{i}}$$

Where *N* represents the number of classes. During model comparison, the precision-recall curve using micro-averaging is employed to evaluate the overall performance. For the F_1_ score, macro-averaging is adopted to assess the performance of each class, revealing the model's balance and robustness across classes, particularly in scenarios with imbalanced class distributions.

S1.2 Regression Metrics Used for ML Valuations

S1.2.1 **R^2^ Score**

The R^2^ score, also known as the coefficient of determination, is a commonly used statistical measure in regression analysis to assess how well the regression model fits the data. It represents the proportion of variance in the actual values that can be explained by the predicted values of the model. The R^2^ score ranges from [-∞, 1], where a value closer to 1 indicates a better fit of the model to the data.

**S1.2.2 RMSE**

The RMSE is another critical performance evaluation metric for regression tasks, measuring the deviation between the predicted and actual values. RMSE is the square root of the mean of the squared residuals and shares the same unit as the target variable. A smaller RMSE indicates smaller prediction errors. Since squaring the errors amplifies the impact of larger deviations, RMSE is highly sensitive to outliers.

**S1.2.3 Violin Plot**

The violin plot is a visualization tool that combines box plots and kernel density estimation to present the data distribution, central tendency, and probability density. It visually displays the statistical summary of the data on a single plot (Fig. S33). The outer shape of the violin plot is formed by a smooth, symmetric curve derived from kernel density estimation, intuitively representing the probability density of the data distribution. A wider section of the plot indicates a higher probability of data occurring within that value range. The interior of the violin plot incorporates conventional box plot elements, where the box boundaries represent the interquartile range, and the white dot within the box indicates the median, reflecting the central tendency of the data.

**S2 Supplementary Figures and Tables**


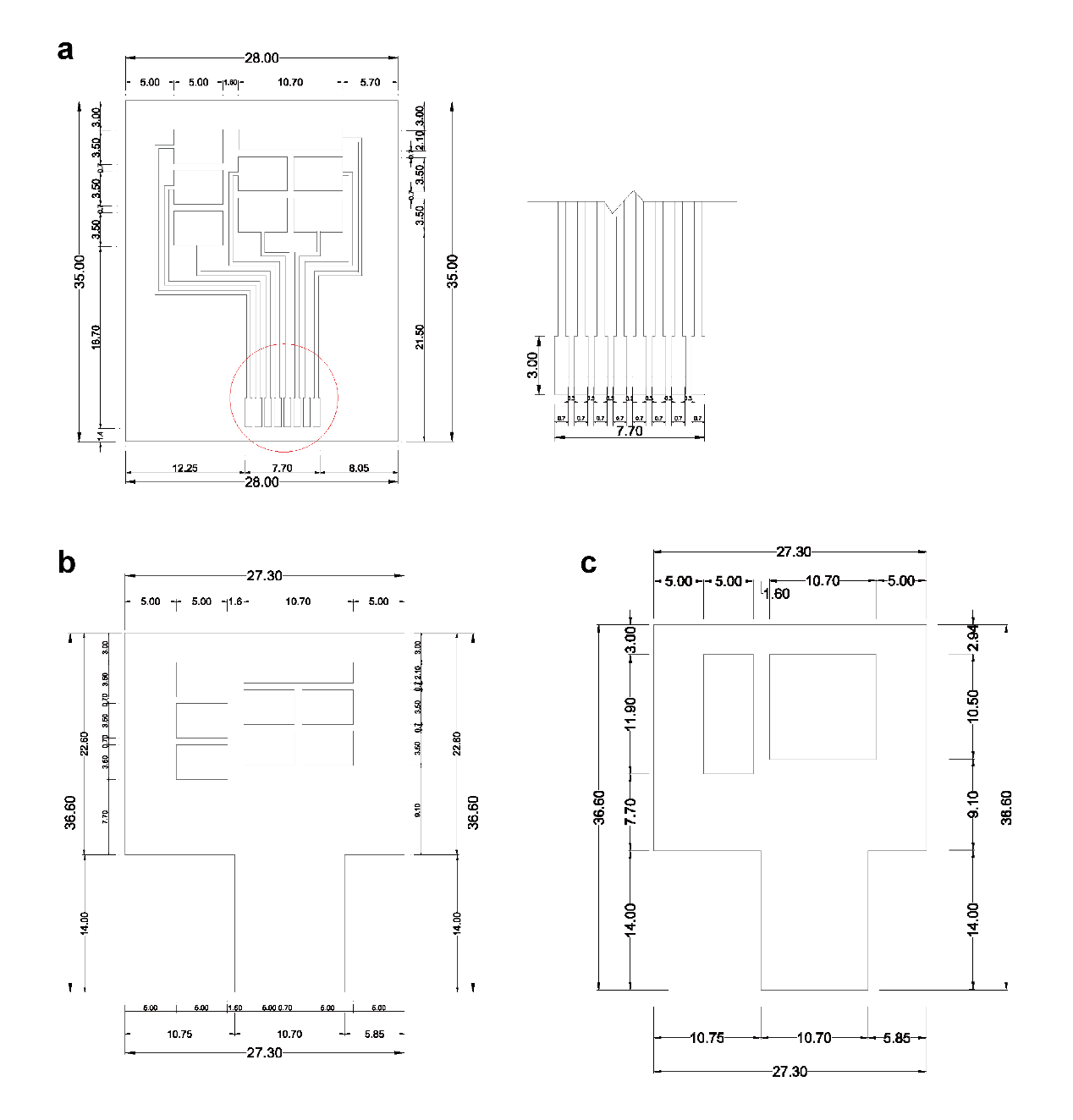


**Fig. S1** Design and detailed dimensions of components used for the assembly of the eMPatch. **a** Design of the screen printing mask used for interconnects and pin assignments. **b** Design of the laser-engraving layout for the MN fixing. **c** Design of the laser-cutting layout for medical tape and dressing film


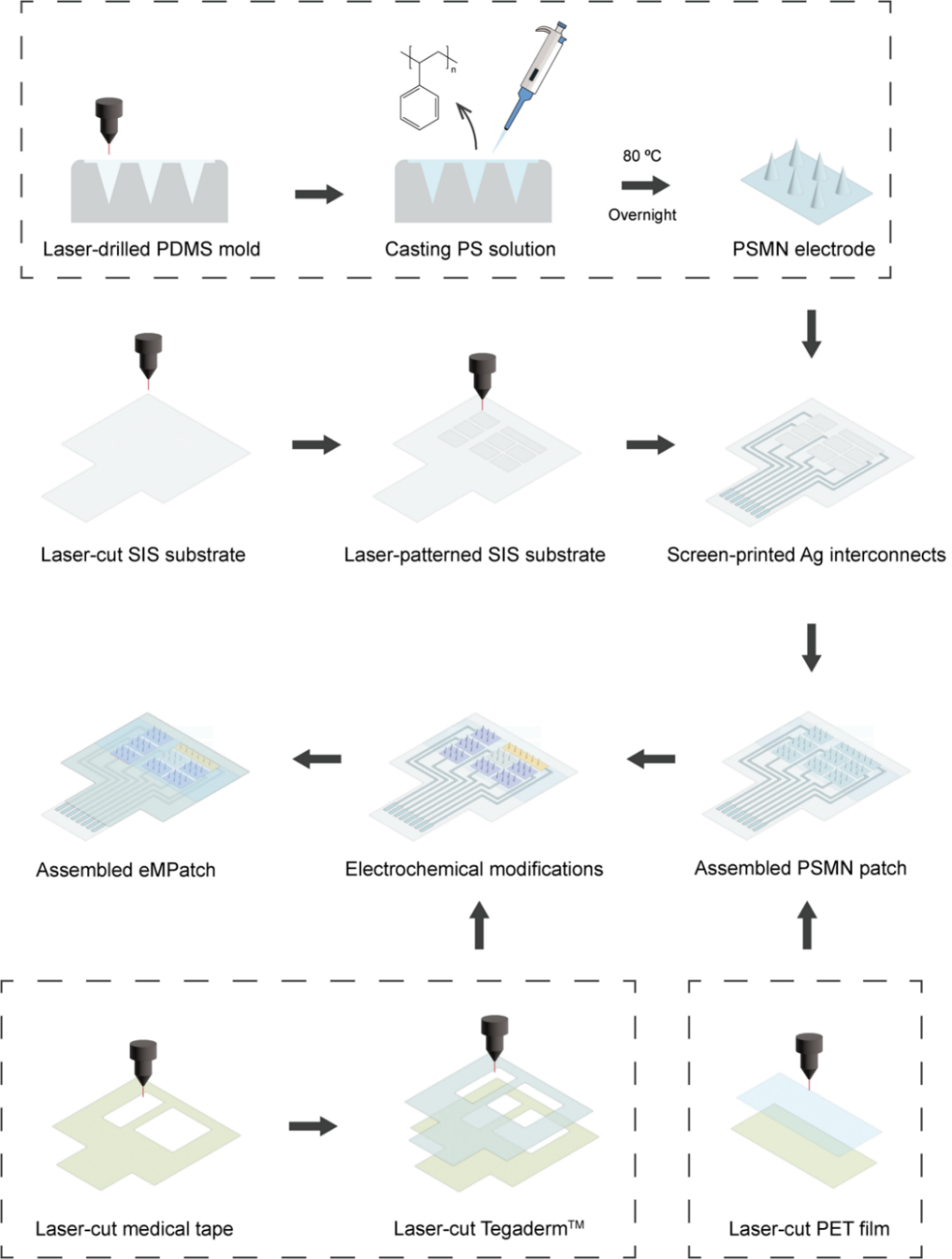


**Fig. S2** Schematics of the fabrication and assembly process of the eMPatch


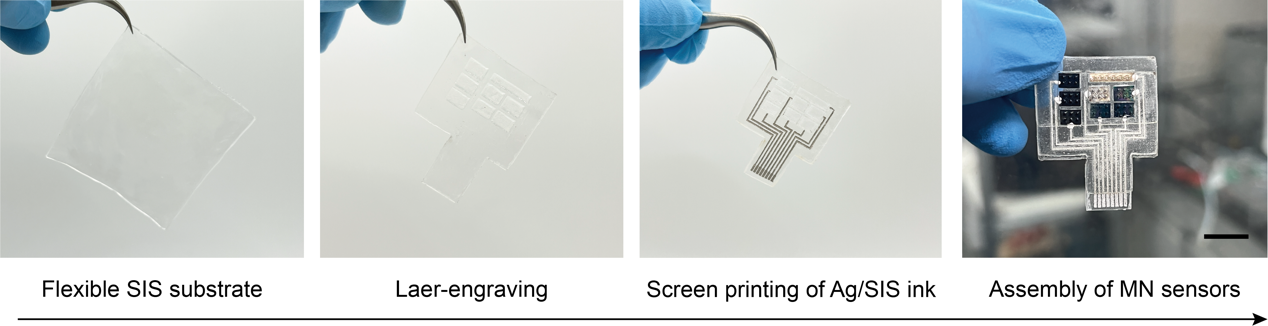


**Fig. S3** Fabrication and assembly process of the eMPatch. Scale bar, 1 cm


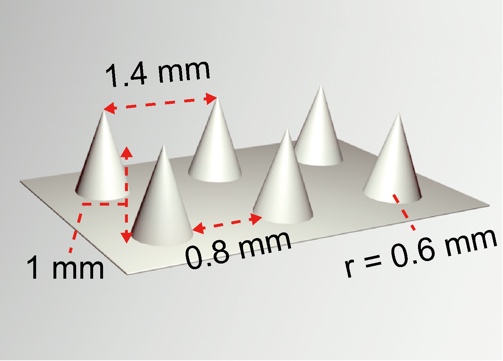


**Fig. S4** Configuration of the MN array with corresponding dimensions


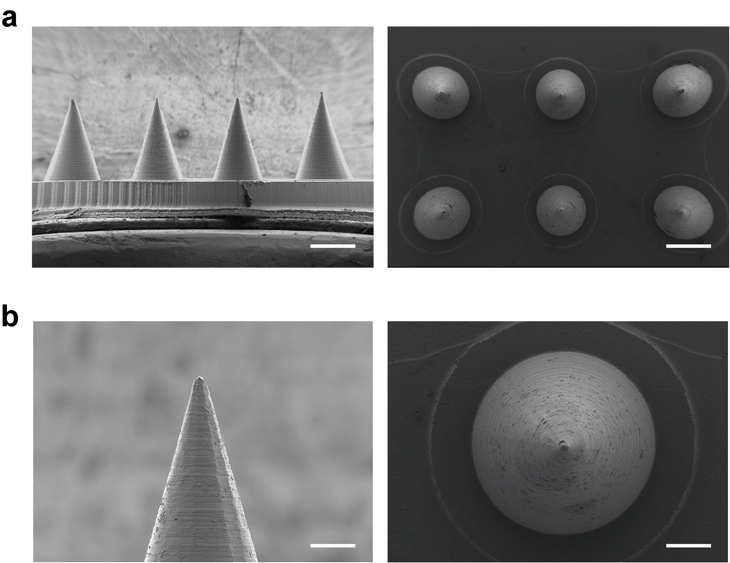


**Fig. S5** SEM images of the MN array. **a** Side view (left) and top view (right) of the MN array. Scale bar, 400 μm. **b** Zoom-in images of the needle tip from side view (left) and top view (right). Scale bar, 100 μm


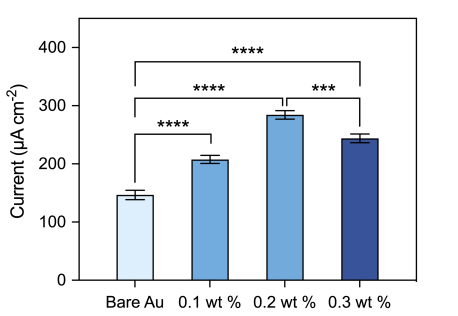


**Fig. S6** Peak current of CV scans of AuMN modified with the EDOT concentrations ranging from 0 to 0.3 wt % in a 5 mM [Fe(CN)_6_]^3-/4-^ solution. Scan rate, 50 mV s^-1^. Error bar indicates SD from three replicates (n = 3)


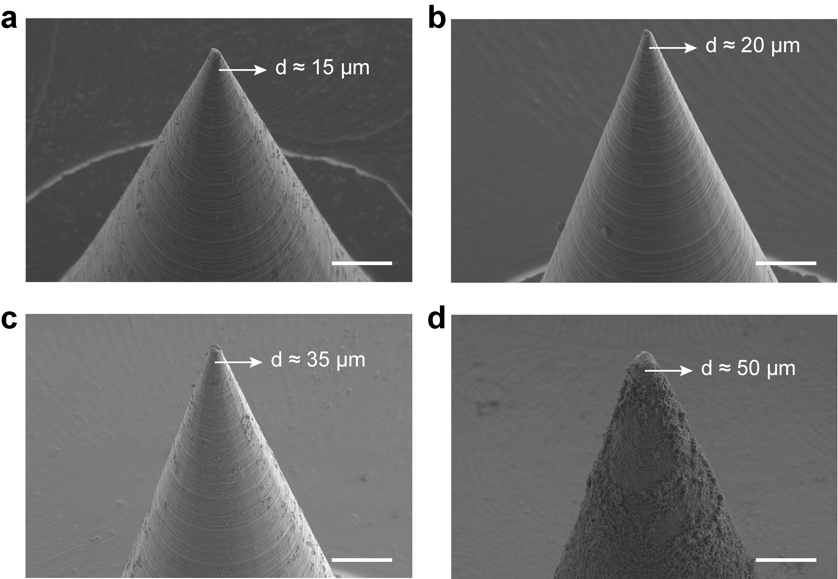


**Fig. S7** SEM images of MN tips after each fabrication step: initial MN (**a**), AuMN (**b**), PEDOT:PSS modified AuMN (**c**), and Prussian Blue decorated AuMN (**d**). Scale bar, 50 μm


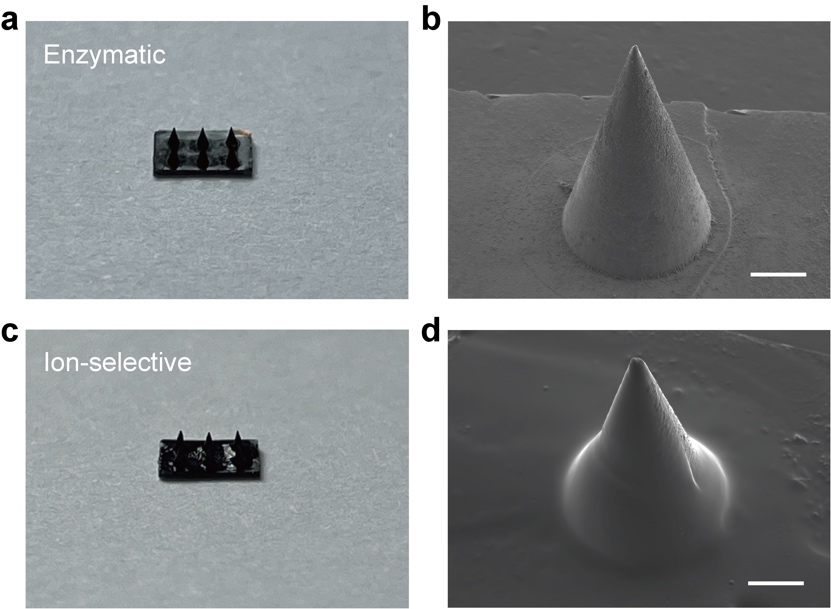


**Fig. S8** Optical and SEM images showing the MN tips after depositing the selective layers. Scale bar, 50 μm


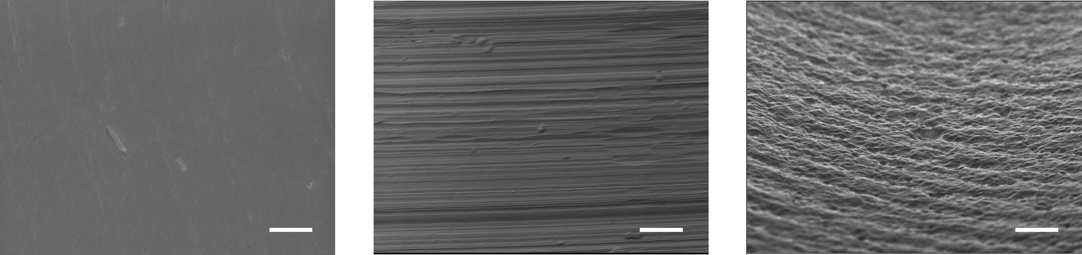


**Fig. S9** SEM images of the Au-sputtered planar electrode (left), AuMN (middle), and PEDOT:PSS modified AuMN (right). Scale bar, 10 μm


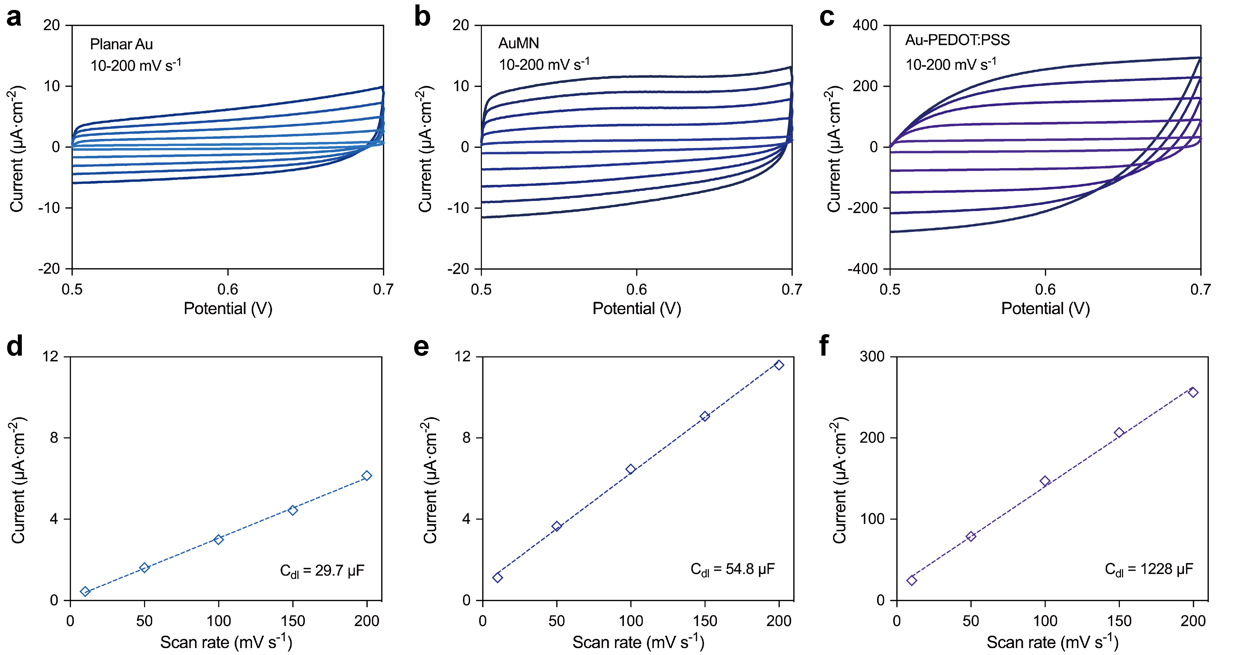


**Fig. S10** CV characterizations of the MN sensors. **a-c** CV measurements of the Au-sputtered planar electrode (**a**), AuMN (**b**), and PEDOT:PSS modified AuMN (**c**) at different scanning rates (10, 50, 100, 150, and 200 mV s^-1^) in the potential range from 0.5 to 0.7 V. **d-f** The corresponding calibration curves indicating the correlation between the oxidation current density at 0.6 V and scan rates of Au-sputtered planar electrode (**d**), AuMN (**e**), and PEDOT:PSS modified AuMN (**f**)


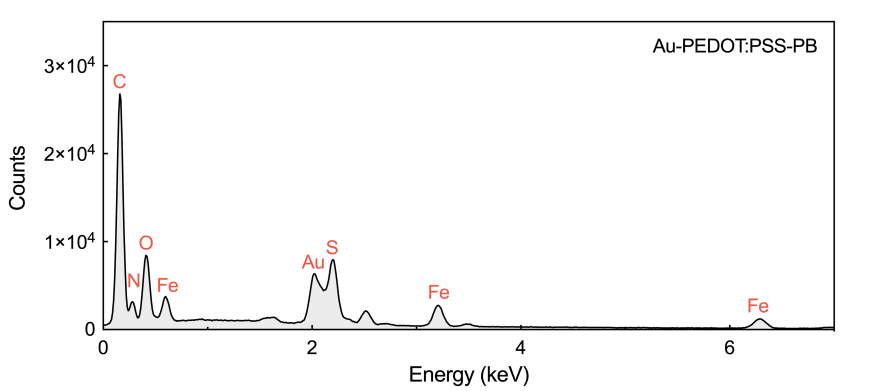


**Fig. S11** EDS elemental spectrum of the as-modified MN sensors


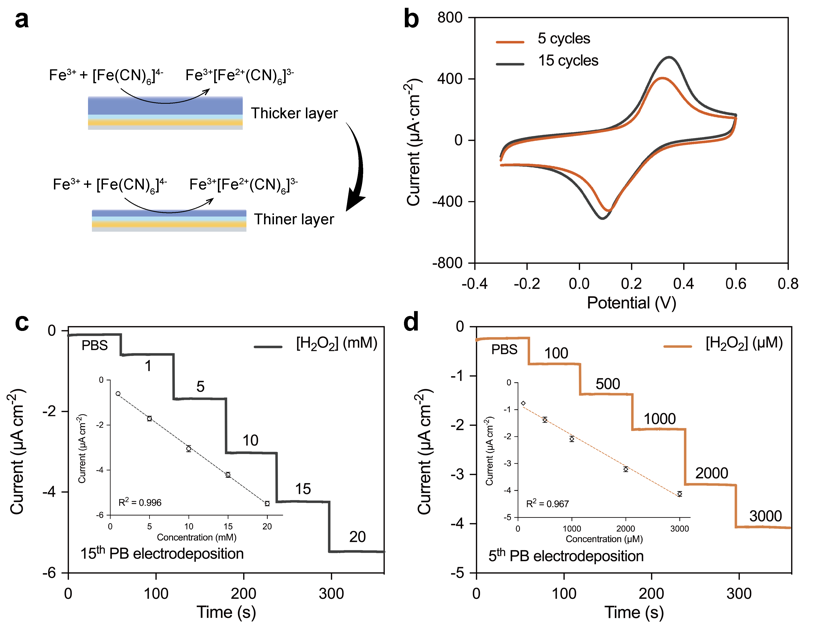


**Fig. S12 a** Schematic showing the modified PB with different thicknesses as the electron transfer mediator for H_2_O_2_ detection. **b** Comparison of the CV curves obtained after 5 cycles and 15 cycles of PB electrodeposition. **c** Chronoamperometric responses of the H_2_O_2_ ranging from 0-20 mM with 15 cycles of PB electrodeposition. Inset, the corresponding calibration curve (n = 3). **d** Chronoamperometric responses of the H_2_O_2_ ranging from 0-3000 μM with 5 cycles of PB electrodeposition. Inset, the corresponding calibration curve. Error bar indicates SD from three replicates (n = 3)


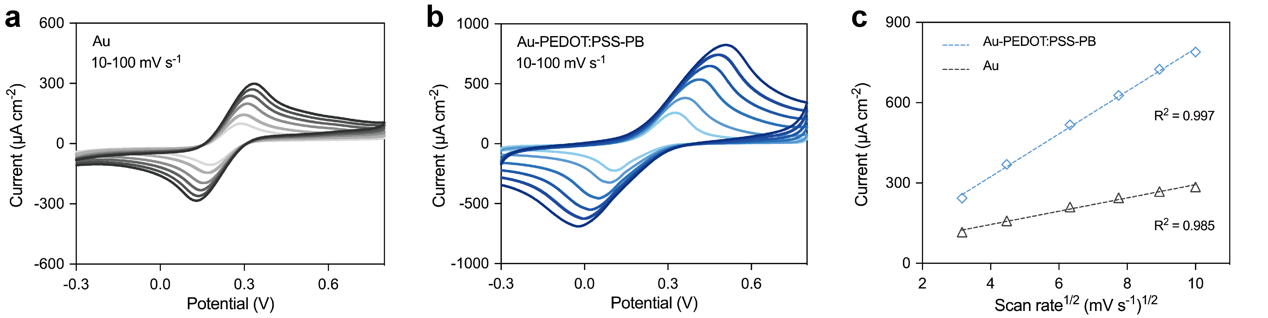


**Fig. S13** Electrochemical characterization of the MN sensor with different scanning rates. **a-b** CV measurements of the bare (**a**) and as-modified conductive MN sensors (**b**) at different scanning rates (10-100 mV s^-1^) in a 5 mM [Fe(CN)_6_]^3-/4-^ solution. **c** The corresponding calibration curves of the square root of scanning rates versus anodic peak current

**Fig. S14** SEM image of the electropolymerized PANI for pH detection. Scale bar, 500 nm


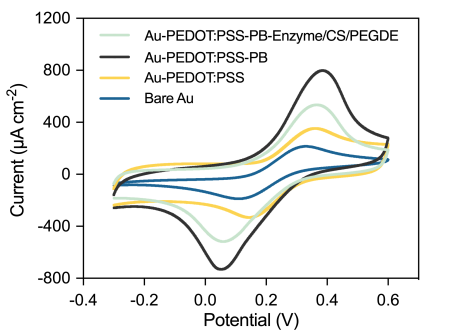


**Fig. S15** CV measurements of the MN working electrode (MN-WE) in a 5 mM [Fe(CN)_6_]^3-/4-^ solution after each modification step: Au, PEDOT:PSS, PB and enzyme/CS/PEGDE network


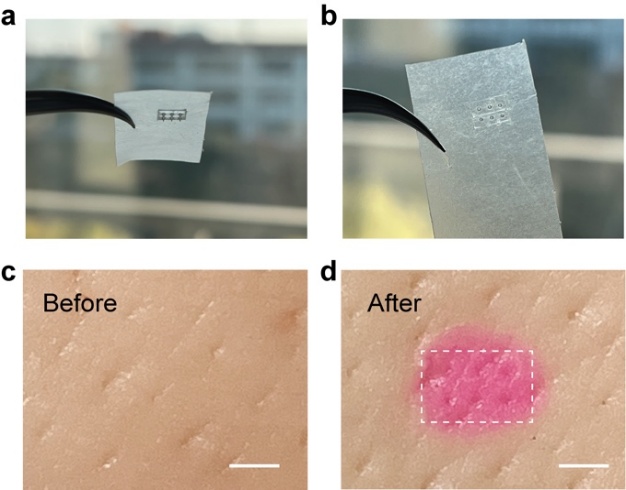


**Fig. S16** Insertion tests of the MN in Parafilm (**a-b**) and in porcine skin (**c-d**)


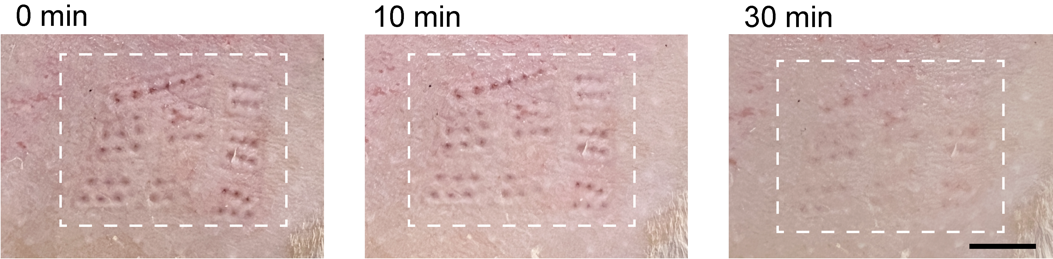


**Fig. S17** Photographs showing the recovery process of 0 min, 10 min, and 30 min after MN insertion for 7 days. Scale bar, 0.5 cm


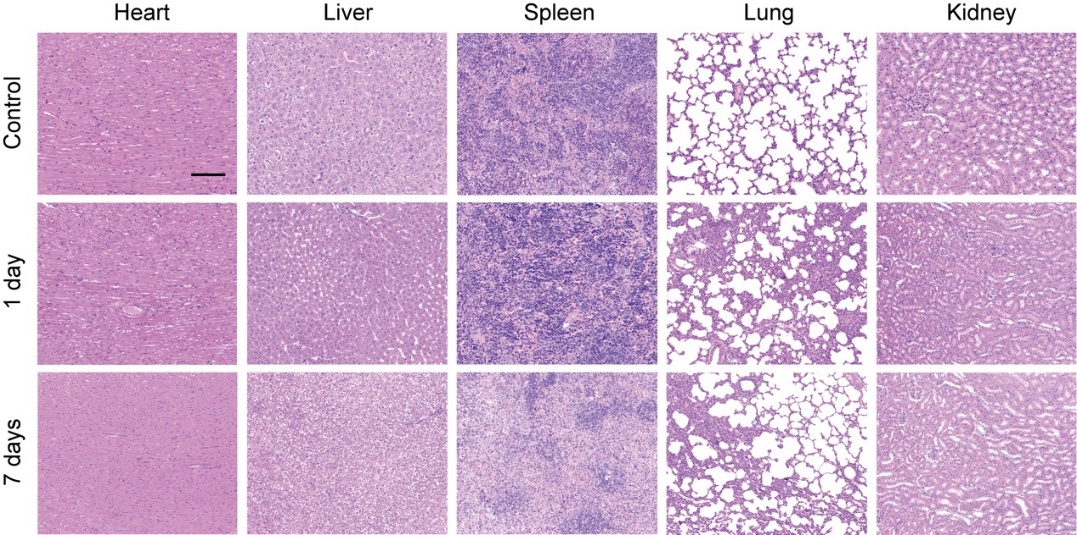


**Fig. S18** H&E staining illustrating the major organs (heart, liver, spleen, lung, and kidney) of rats wearing the eMPatch for 0, 1, and 7 days. Scale bar, 100 μm


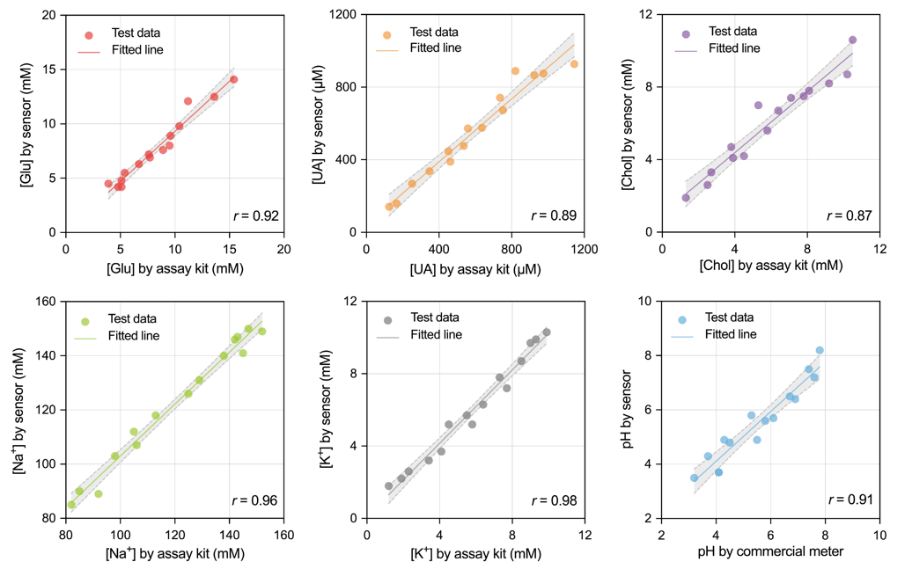


**Fig. S19** Validation of the MN sensors against commercial colorimetric assay kits from 15 analytes replicates in the artificial ISF with elevated concentrations (n = 15). Dash lines indicate a 95% confidence interval


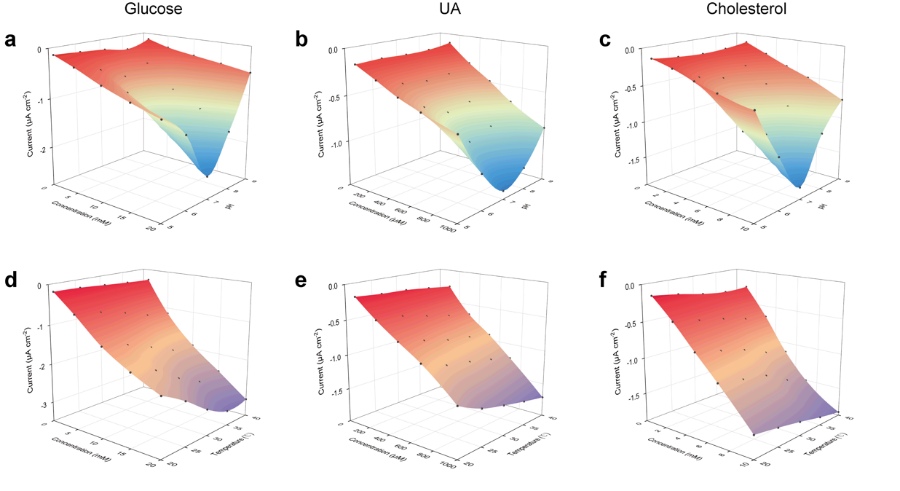


**Fig. S20 a-f** Influence of pH (**a-c**) and temperature (**d-f**) on the amperometric responses of enzymatic sensors


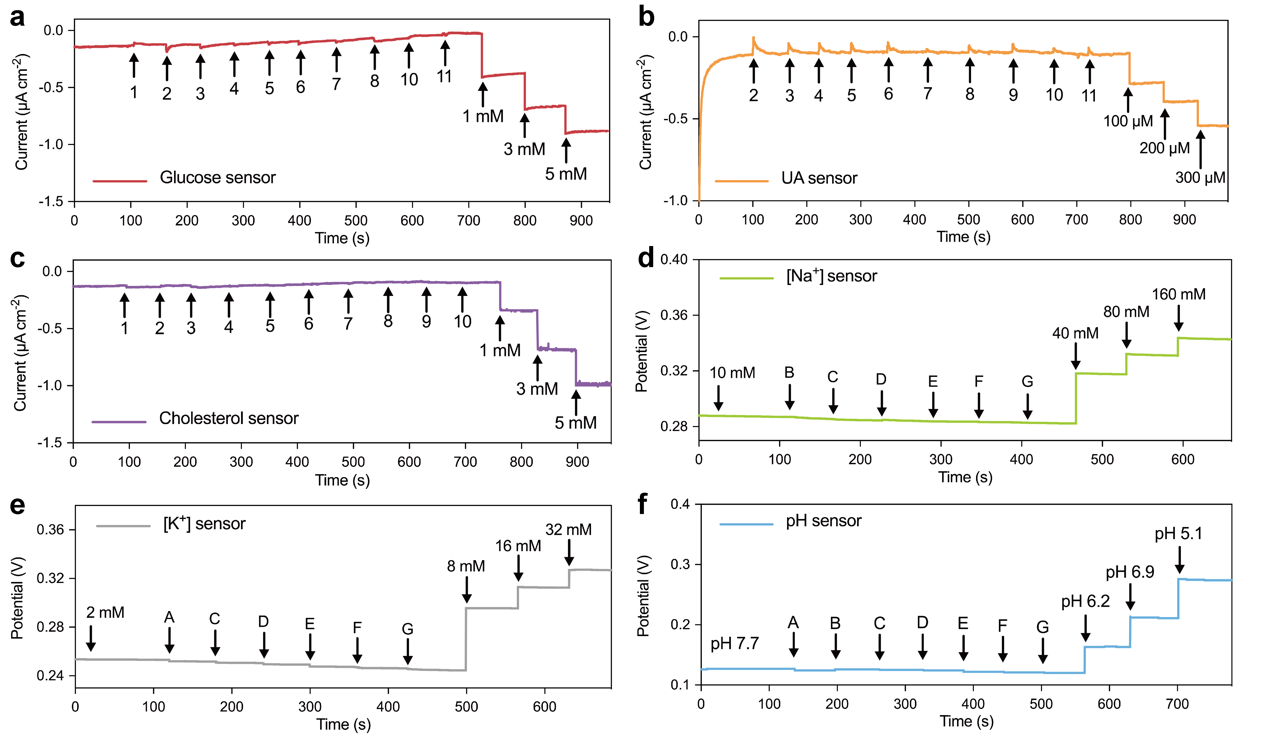


**Fig. S21** Selectivity tests of the eMPatch. **a-c** Selectivity characterization for the glucose (**a**), UA (**b**), and cholesterol sensors (**c**) by chronoamperometric measurements in the presence of commonly found interferences in ISF (1: UA, 2: ascorbic acid, 3: sodium chloride, 4: potassium chloride, 5: histidine, 6: tryptophan, 7: aspirin, 8: acetaminophen, 9: glucose, 10: lactate, 11: cholesterol). **d-f** Selectivity characterization for the Na^+^ (**d**), K^+^ (**e**), and pH sensors (**f**) by OCP measurements in the presence of commonly found interferences in ISF (A: NaCl, B: KCl, C: MgCl_2_, D: CaCl_2_, E: glucose, F: UA, G: cholesterol)


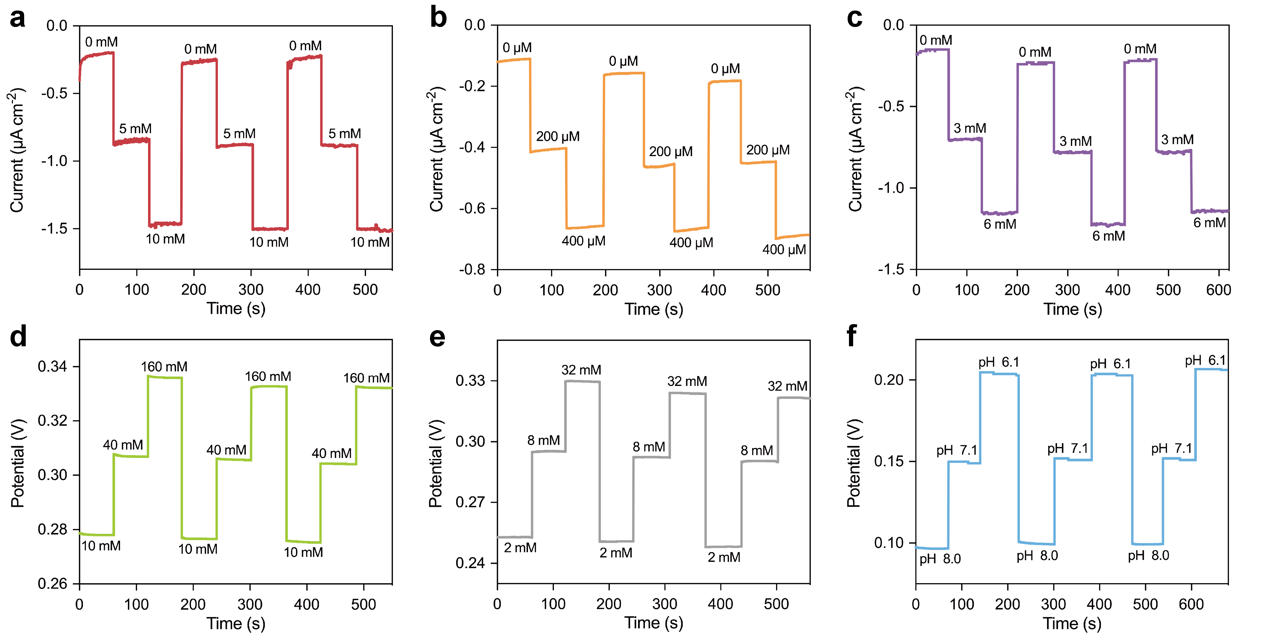


**Fig. S22** **a-f** Repeatability tests over three cycles of stepwise measurements (**a-c**: glucose, UA, cholesterol; **d-f**: Na^+^, K^+^, pH)


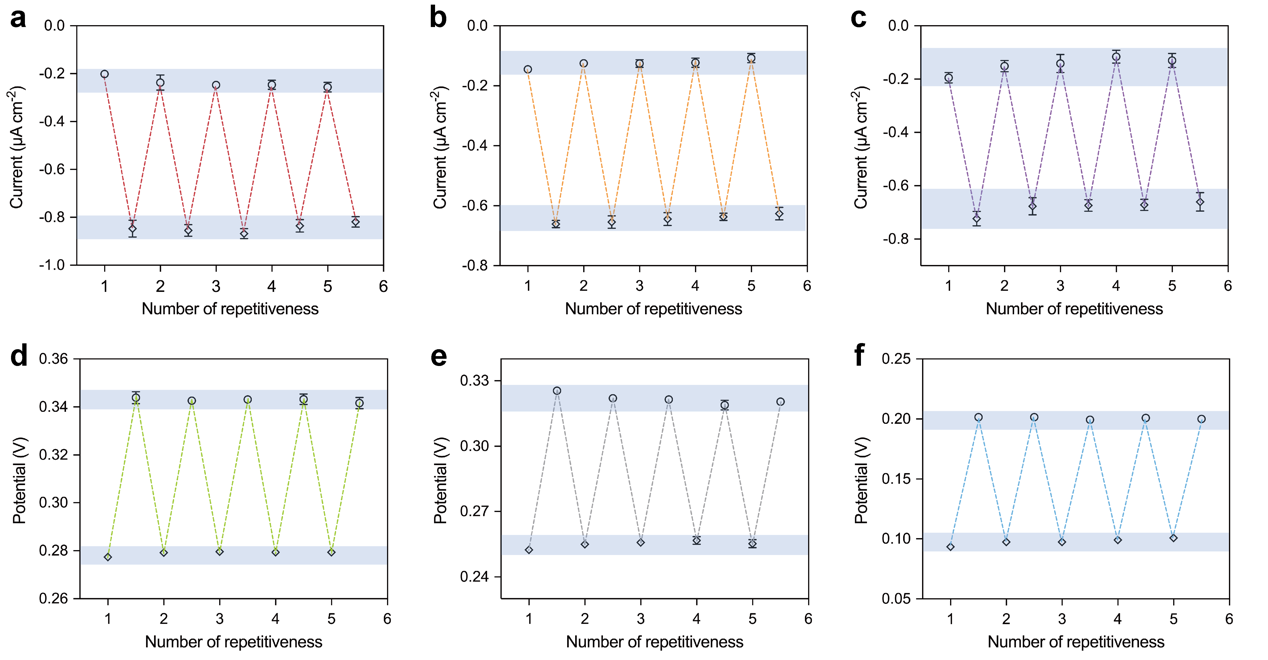


**Fig. S23 a-f** Repeatability tests over five cycles measurements of low-to-high concentrations using three independent eMPatches (**a-c**: glucose, UA, cholesterol; **d-f**: Na^+^, K^+^, pH). Error bar indicates SD from three replicates (n = 3)


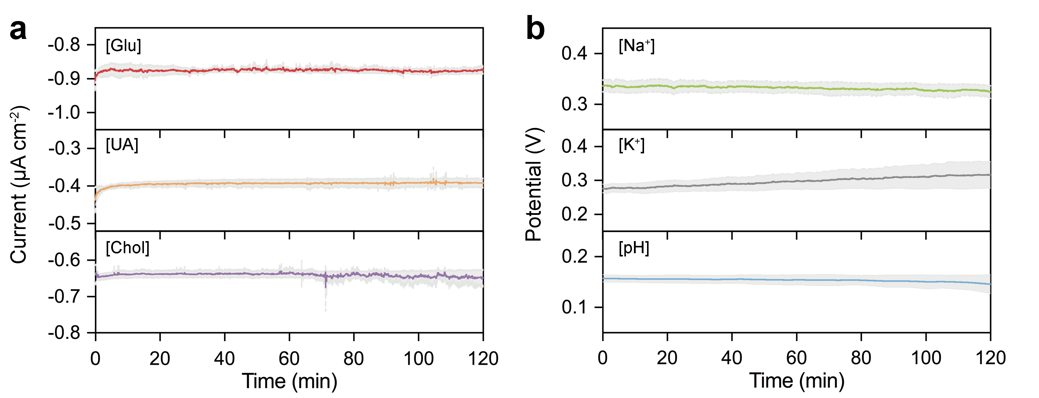


**Fig. S24** Continuous monitoring of targeted biomarkers using the MN sensors over a 120-min period. **a** Amperometric responses of glucose (5 mM), UA (200 μM), and cholesterol (3 mM). **b** Potentiometric responses of Na⁺ (140 mM), K⁺ (4 mM), and pH (7.0). The solid lines represent the mean signal values, and the gray area denotes the SD from three sensors (n = 3)


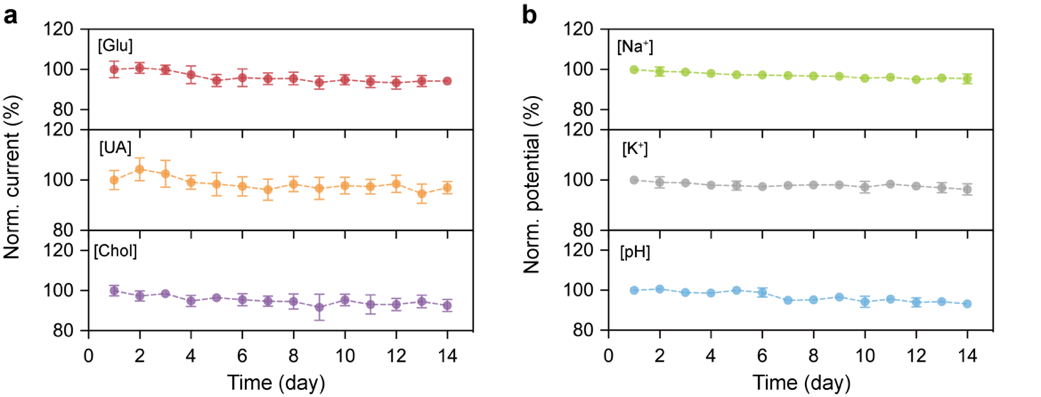


**Fig. S25 a-b** Shelf-life stability of the eMPatch after 0-14 days of storage. Error bar indicates SD from three replicates (n = 3)


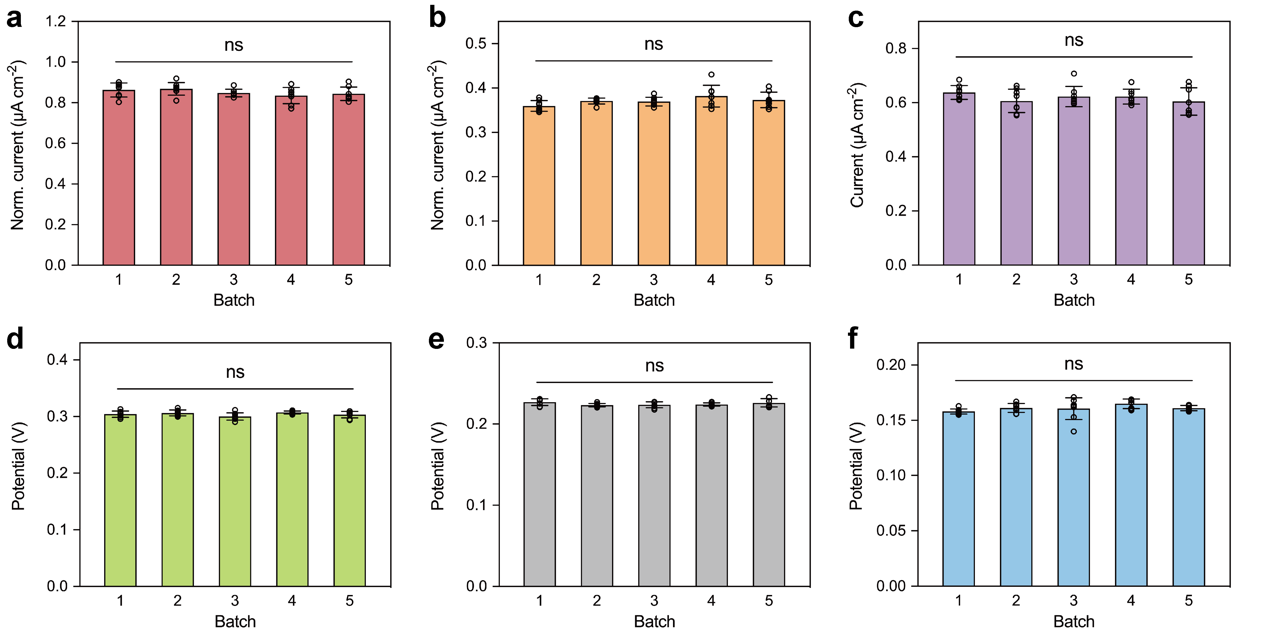


**Fig. S26 a-f** Reproducibility tests of the eMPatch. The batch-to-batch reproducibility tests were performed by comparing the electrochemical responses of independently fabricated sensors from different fabrication batches under identical experimental conditions. The responses of glucose (**a**), uric acid (UA) (**b**), cholesterol (**c**), Na⁺ (**d**), K⁺ (**e**), and pH (**f**) sensors were measured in aISF at fixed analyte concentrations. Error bar indicates SD from eight replicates (n = 8)


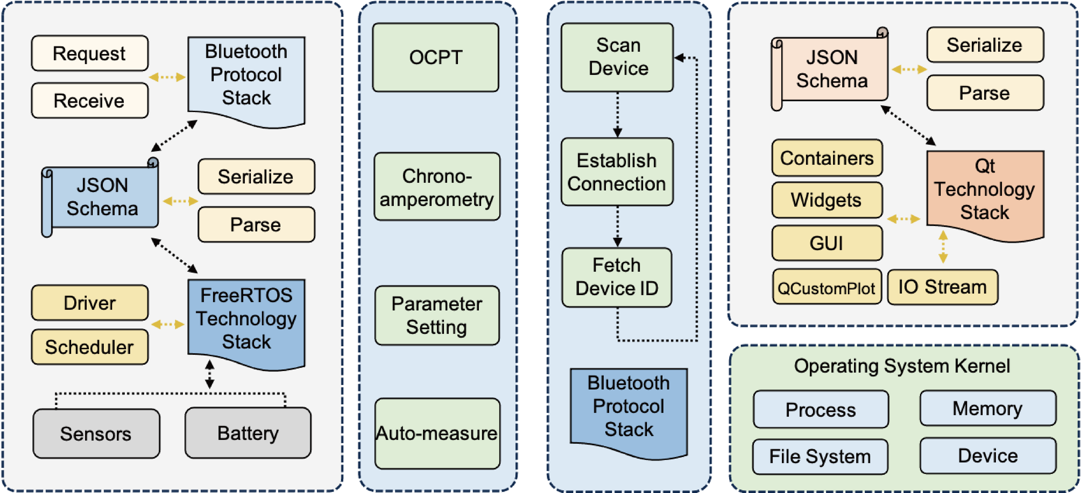


**Fig. S27** Detailed firmware (left) and software (right) block diagram of the electrochemical electronic system


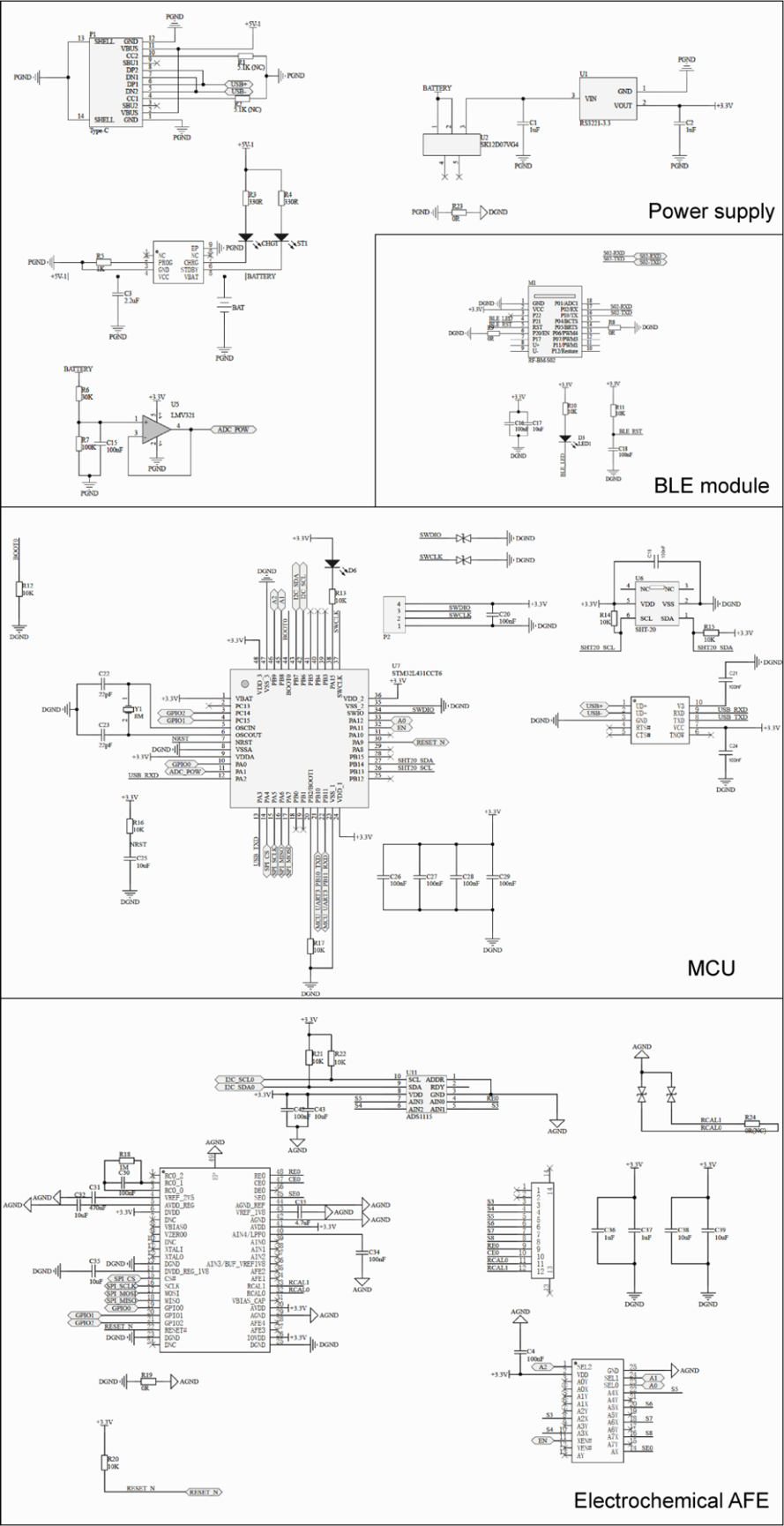


**Fig. S28** Detailed circuit diagram of the electrochemical electronic system for wearable application


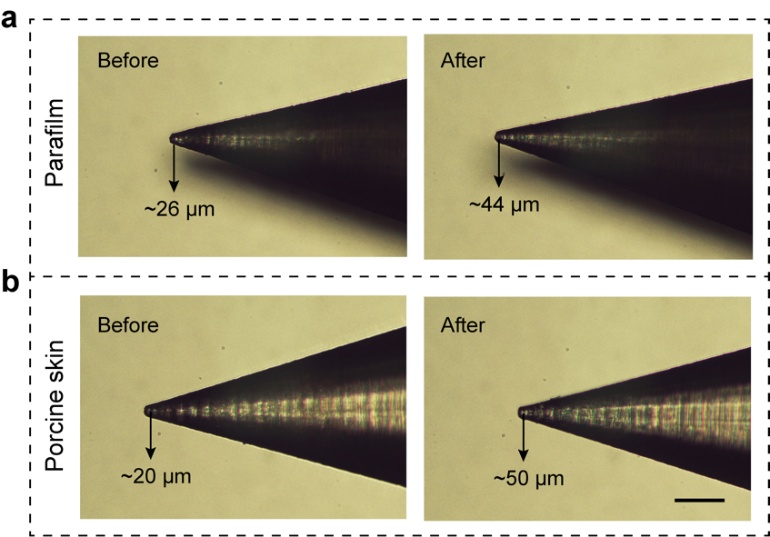


**Fig. S29** Microscopic images showing the MN tips before and after the insertion tests on Parafilm (**a**) and porcine skin (**b**). Scale bar, 200 μm


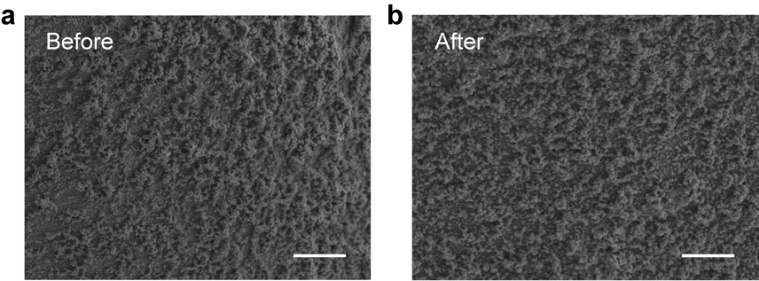


**Fig. S30** SEM images of the functionalized MN surface before (a) and after (b) insertion. Scale bar, 10 μm


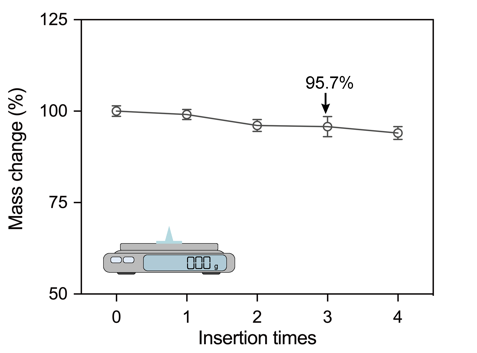


**Fig. S31** Mass changes of a single MN after multiple insertions


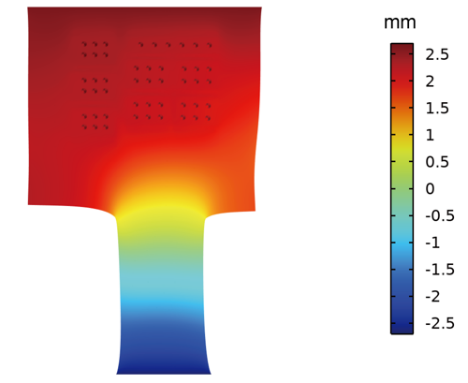


**Fig. S32** Finite element simulation illustrating the displacement of the eMPatch without the PET backing. A horizontal strain percentage of 15% is applied to the eMPatch


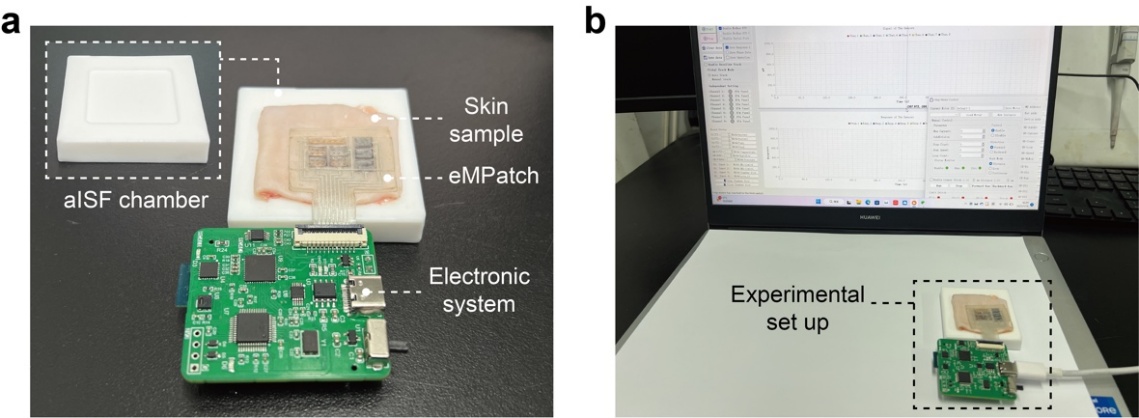


**Fig. S33** Optical images of the ex vivo experimental setup. **a** The ex vivo experimental setup consists of an aISF chamber, a rat skin sample, and the electronic system. **b** Photo showing the benchtop software and the experimental setup


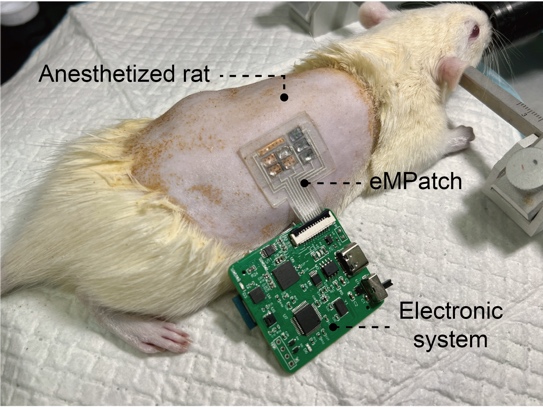


**Fig. S34** Optical image of the eMPatch on the anesthetized SD rat


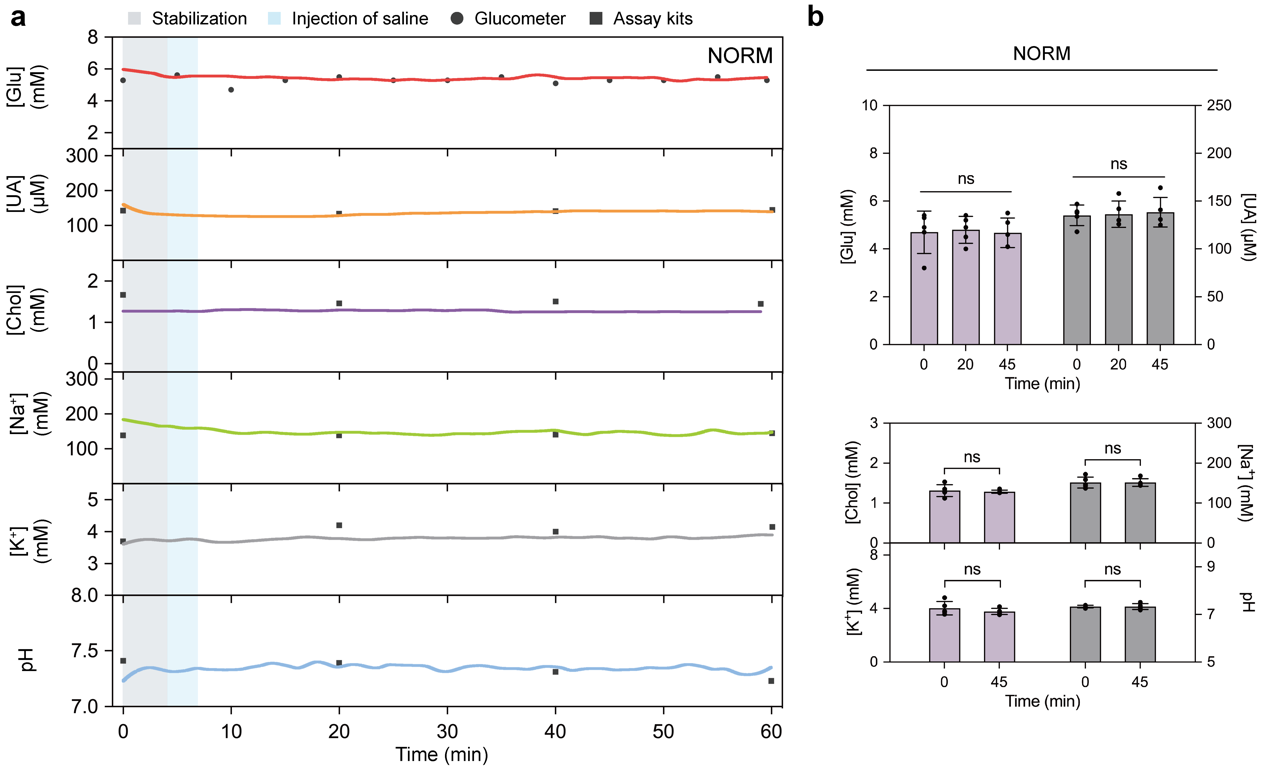


**Fig. S35** In vivo evaluation of the eMPatch on rats of the NORM group. **a** Continuous monitoring of multiplexed biomarkers in the NORM group for an hour. **b** Comparison of the targeted biomarkers in ISF in the stage of: after overnight fasting, 20 min, and 45 min after the saline treatment. Error bar indicates SD from five rats (n = 5). ns, *P*  > 0.05. One-way ANOVA with Tukey's test


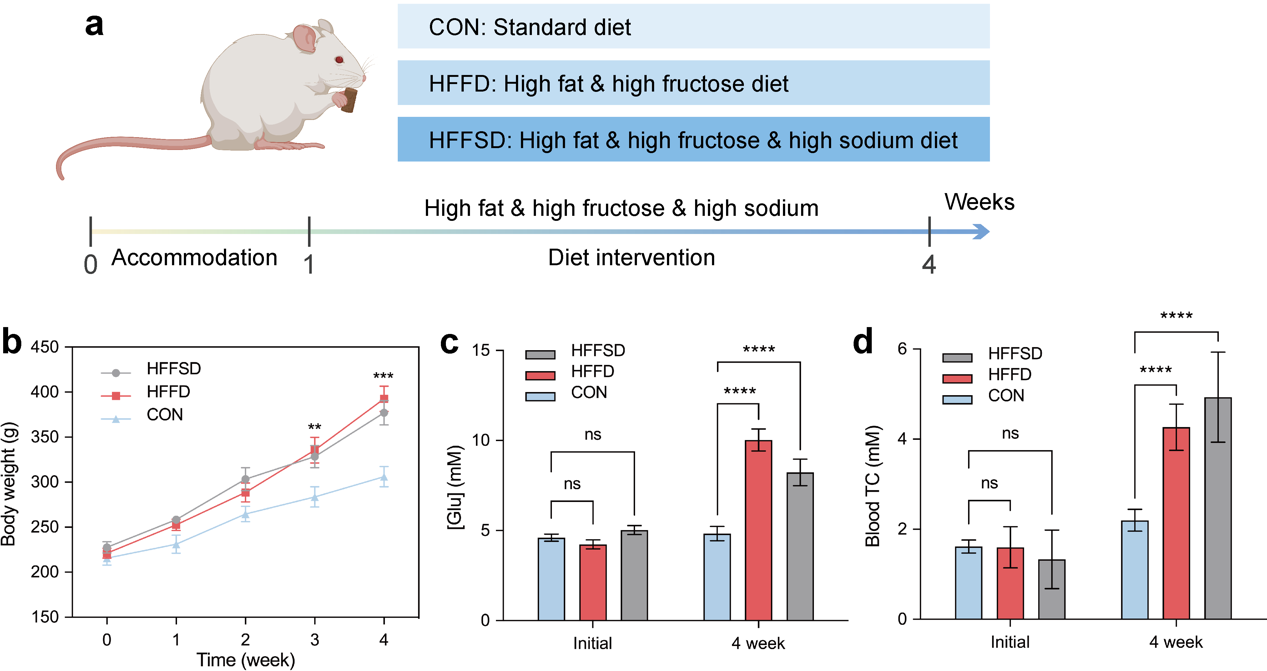


**Fig. S36** Validation of the rat models. **a** Schematic showing the design of the rat models: CON, HFFD, and HFFSD. **b-d** Effects of different diets on body weight gain (**b**), blood glucose (**c**), and blood total cholesterol (TC) (**d**) levels in the CON, HFFD, and HFFSD. The significant elevation of body weight, blood glucose, and blood TC indicated the successful establishment of rat models. Error bar indicates SD from three replicates (n = 3). ^**^*P* < 0.01, ^***^*P* < 0.001, ^****^*P* < 0.0001, ns, *P*  > 0.05. One-way ANOVA with Dunnett's test


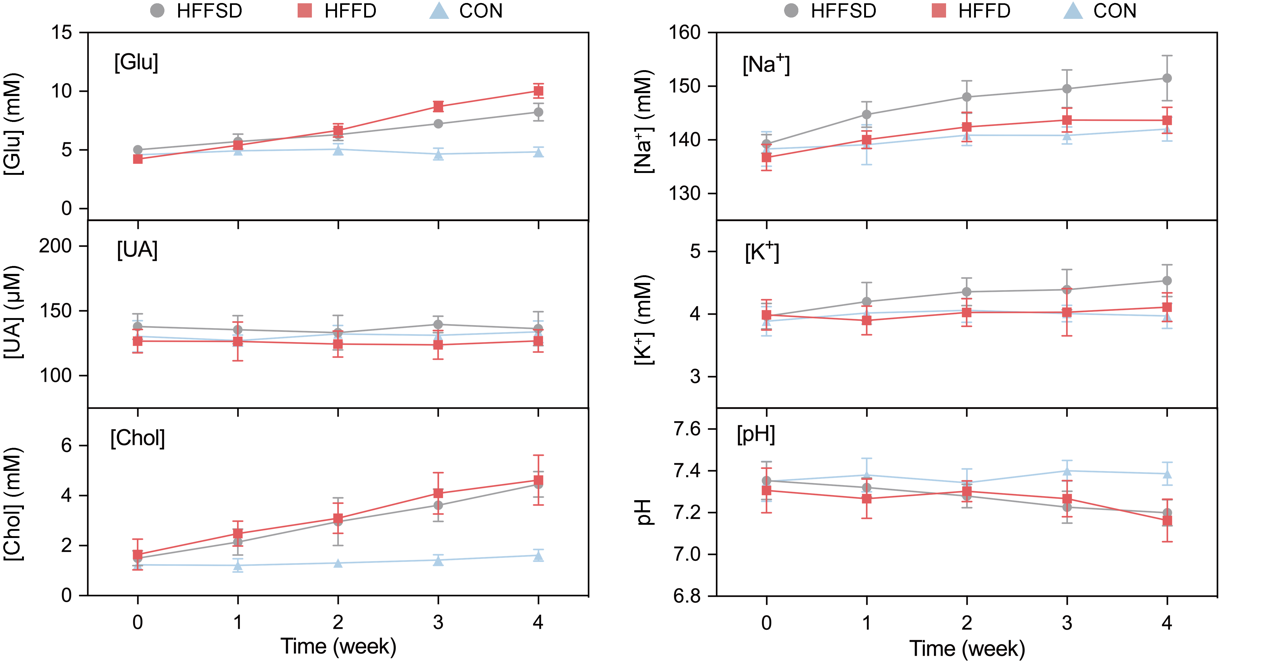


**Fig. S37** Long-term monitoring of multiplexed biomarkers using the eMPatch in rat groups received different diets for 4 weeks. Error bar indicates SD from three replicates (n = 3)


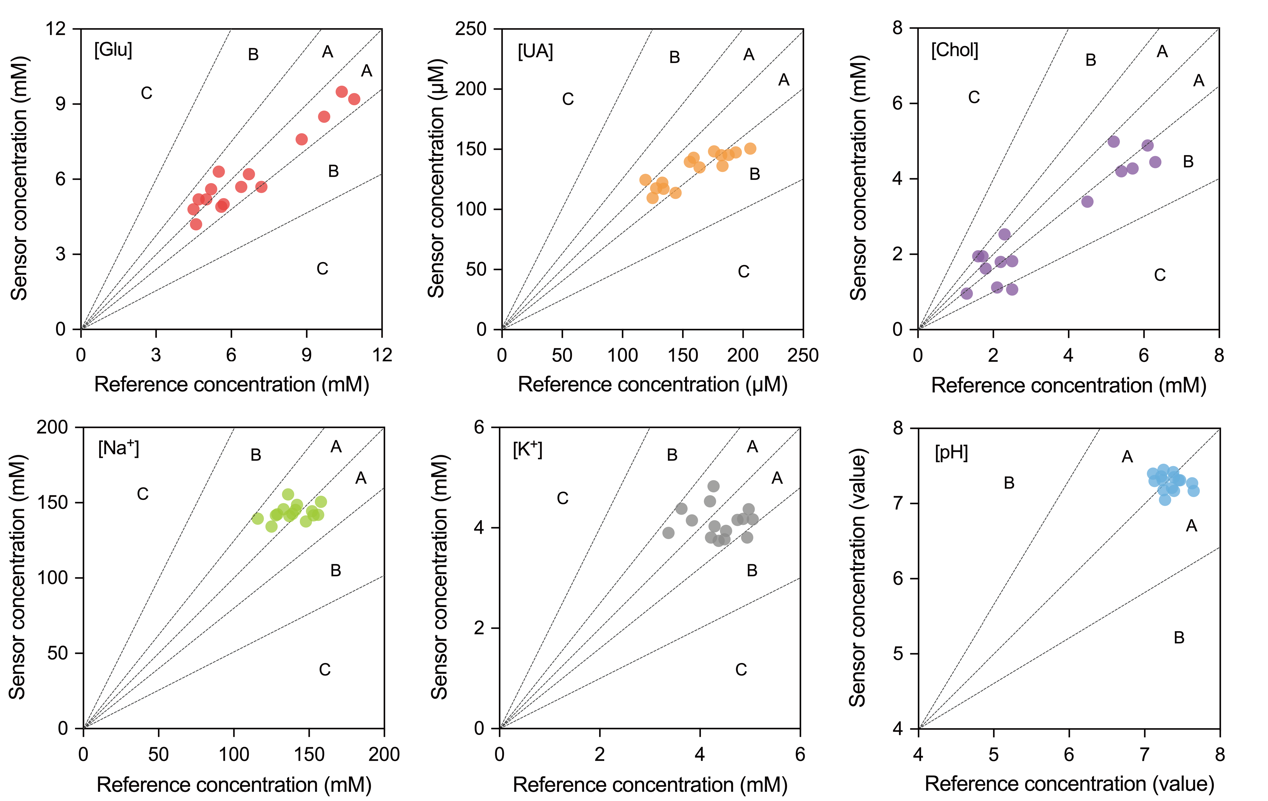


**Fig. S38** Error grid analysis of the collected data using the eMPatch. The readouts of the biosensor from rat groups of CON, HFFD, and HFFSD were validated against commercial reference measurements (glucometer and colorimetric assay kits). Regions A, B, and C indicate sensing errors < 20%, 20%-50%, and > 50%, respectively


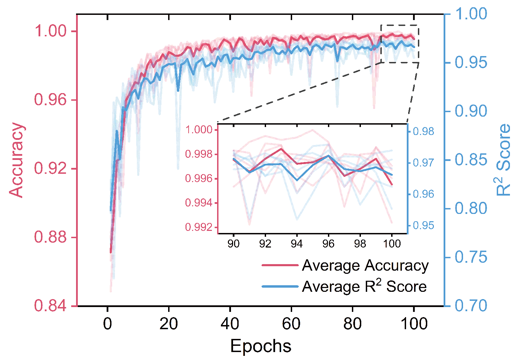


**Fig. S39** Validation curves of classification accuracy and regression R^2^ score under 5-fold cross-validation. The lighter curves represent the results of each fold, while the darker curves show the averaged results across all 5 folds


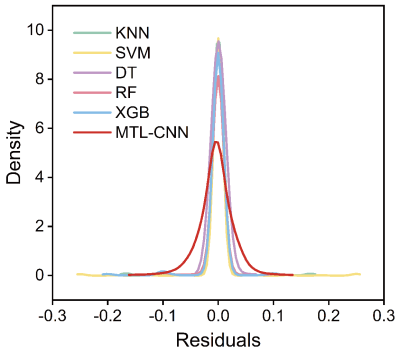


**Fig. S40** Residual distributions of different ML models used for health assessment. KNN, K-Nearest Neighbors; SVM, Support Vector Machine; DT, Decision Tree; RF, Random Forest; XGBoost, Extreme Gradient Boosting


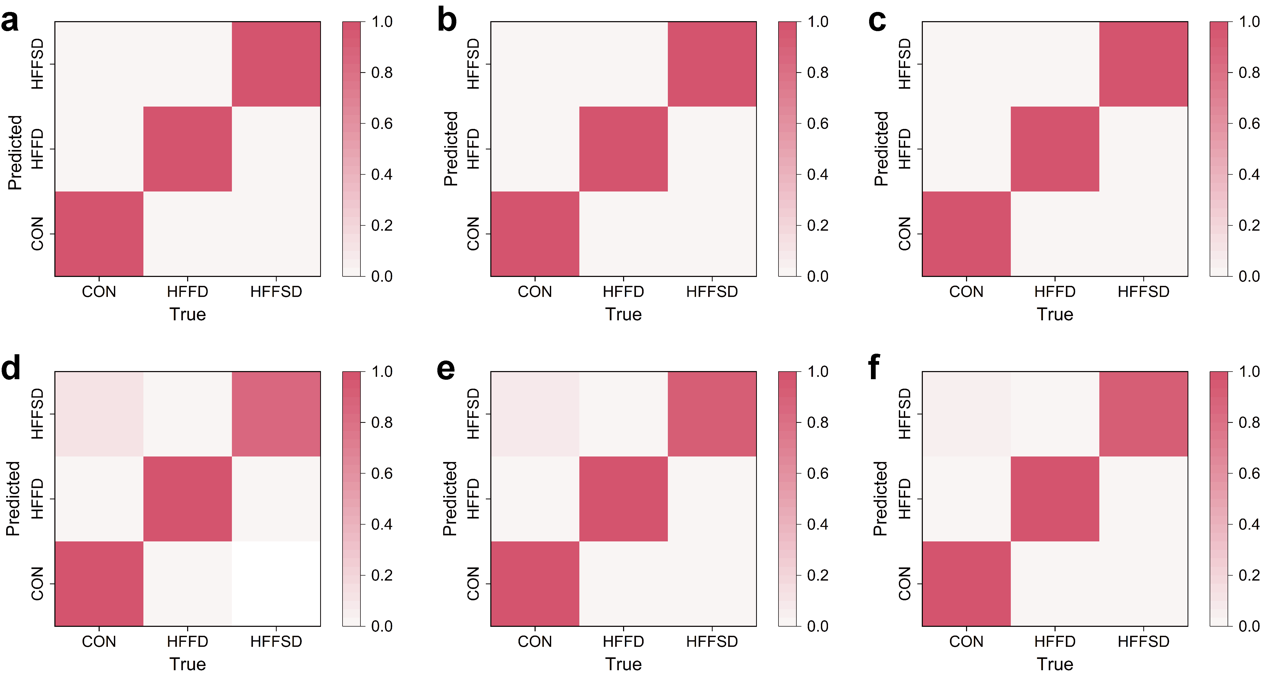


**Fig. S41 a-f** Confusion matrix describing the classification accuracy of the MTL-CNN (**a**), KNN (**b**), SVM (**c**), DT (**d**), RF (**e**), and XGBoost (**f**) for rat groups with different health conditions in the validation dataset


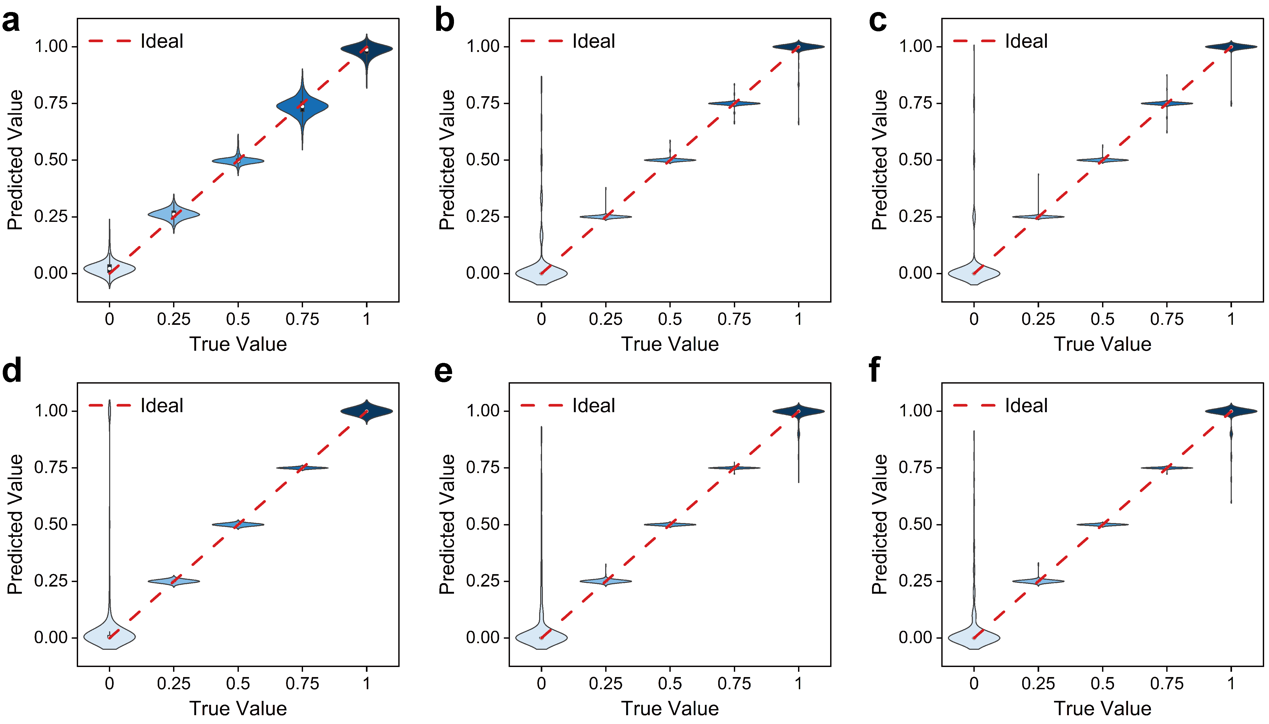


**Fig. S42 a-f** Violin plots depicting true versus predicted value of different ML models. The distribution of health degrees evaluated by the MTL-CNN (**a**), KNN (**b**), SVM (**c**), DT (**d**), RF (**e**), and XGBoost (**f**) in the validation dataset


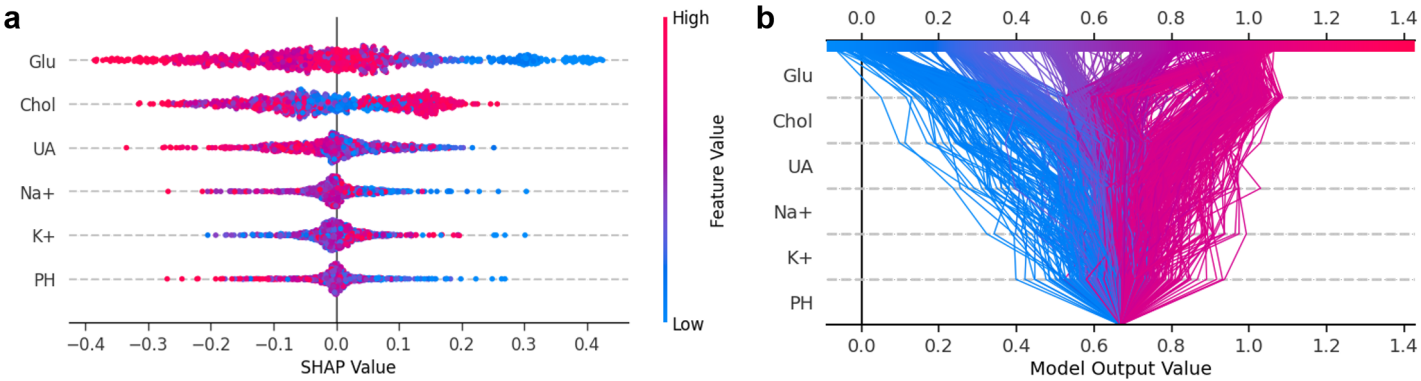


**Fig. S43** SHapley Additive exPlanations (SHAP) analyses of the regression task. **a** The SHAP summary plot with respect to the MTL-CNN model for the regression task based on 1,000 samples. **b** SHAP decision plot explaining how the MTL-CNN model determines the health degrees using molecular features

| CAD designs |  |  |  |  |
| --- | --- | --- | --- | --- |
| Schematic diagrams | 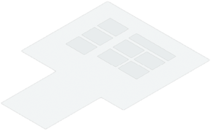 | 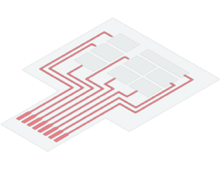 | 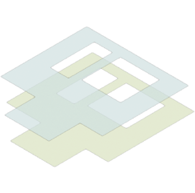 | 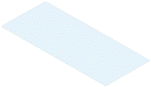 |
| Materials | SIS elastomer | PET tape | Medical tape  Dressing film | PET film |
| Laser mode | Black: cutting  Green: engraving | Black: cutting  Green: engraving | Cutting | Cutting |
| Power  (25 W, 100%) | Black: 55%  Green: 8% | Black: 12%  Green: 15% | 40%  20% | 20% |
| Speed  (mm s^-1^) | Black: 150  Green: 50 | Black: 100  Green: 100 | 100 | 50 |
| Pitch (mm) | 0.03 | 0.05 | 0.05 | 0.05 |

**Table S1** Optimized parameters for the fabrication of eMPatch

**Table S2** Accuracy of the eMPatch compared with standard reference methods

| **Biomarkers** | **Reference method** | **R^2^** | **Mean relative error (%)** | **Accuracy within 20% (%)** | **Accuracy within 50% (%)** |
| --- | --- | --- | --- | --- | --- |
| Glucose | Glucometer | 0.91 | 6.3 | 93.3 | 100 |
| Uric acid | Colorimetric assay | 0.77 | 12.5 | 60 | 100 |
| Cholesterol | Colorimetric assay | 0.88 | 13.2 | 46.7 | 93.3 |
| Na^+^ | Colorimetric assay | / | 7.9 | 100 | 100 |
| K^+^ | Colorimetric assay | / | 11.5 | 93.3 | 100 |
| pH | Micro pH meter | / | 2.5 | 100 | 100 |

The percentage accuracy within a 20% error margin was calculated as the proportion of data points whose relative error between the eMPatch and the reference method was ≤20%. The mean relative error (MRE) was calculated as the average of the absolute relative errors across all measurements.

**Table S3** Detailed information of the MTL-CNN model

| **Module** | **Layer** | **Input Size** | **Output Size** | **Kernel Size** | **Stride** | **Padding** |
| --- | --- | --- | --- | --- | --- | --- |
| Shared  Block | Conv1 | 6×6×1 | 6×6×32 | 5×5 | 1 | 2 |
|  | MaxPool1 | 6×6×32 | 5×5×32 | 2×2 | 1 | 0 |
|  | Conv2 | 5×5×32 | 5×5×96 | 3×3 | 1 | 1 |
|  | MaxPool2 | 5×5×96 | 4×4×96 | 2×2 | 1 | 0 |
|  | Conv3 | 4×4×96 | 4×4×128 | 3×3 | 1 | 1 |
|  | MaxPool3 | 4×4×128 | 3×3×128 | 2×2 | 1 | 0 |
|  | Conv4 | 3×3×128 | 3×3×128 | 3×3 | 1 | 1 |
|  | Conv5 | 3×3×128 | 3×3×256 | 3×3 | 1 | 1 |
|  | MaxPool4 | 3×3×256 | 2×2×256 | 2×2 | 1 | 0 |
| Classification Branch | Linear1 | 1024 | 128 | \ | \ | \ |
|  | Linear2 | 128 | 3 | \ | \ | \ |
| Regression  Branch | Linear1 | 1024 | 128 | \ | \ | \ |
|  | Linear2 | 128 | 1 | \ | \ | \ |

**Table S4** Performance comparison of the eMPatch with state-of-the-art wearable sensing platforms

| **Wearable platform** | **Biological sample(s)** | **Materials** | **Analytes** | **Sensitivity and limit-of-detection (LOD)** | **Application** | **Advantages and trade-offs** |
| --- | --- | --- | --- | --- | --- | --- |
| Wearable biochip [S1] | Sweat | Electrocatalytically active MIP | Phenylalanine and chloride | 10-300 μM: 1.4 nA μM^−1^ and LOD of 4.7 μM  300-1000 μM: 0.27 nA μM^−1^ | Exercise metabolism | Integrated wearable chip with Phenylalanine-imprinted MIP;  Single-time-point measurements (not continuous monitoring) |
| Printable MIP-based biosensor [S2] | Sweat | Core–shell, MIP/NiHCF  nanoparticles | Ascorbic acid, creatinine, and tryptophan | - | Nutrition and drug monitoring | Scalable wearable biosensor with high stability and long shelf-life;  Lack of long-term validation |
| Smart contact lens [S3] | Tear | Screen-printed  PB-modified  carbon | Glucose | LOD: 0.02 mM | Diabetes | Pre-clinical stage with pilot human testing;  Lack of long-term and continuous monitoring |
| SEB sensor [S4] | Solid-state epidermal biomarkers | Ionic conductive and electronically conductive hydrogel bilayer | Lactate and cholesterol | Lactate: 93.8 nA nmol^−1^ cm^−2^  Cholesterol: 201.34 nA nmol^−1^ cm^−2^ | Daily activities | Solid analytes monitoring, human testing;  Limited operational time |
| PillTrek [S5] | Intestinal fluids | Inkjet-printed gold  nanoparticles | Glucose, pH, serotonin, and  ionic strength | Glucose: −2.78 nA mM^−1^  pH: 54.84 mV per pH  Ionic strength: 7.15 mS mM^−1^  LOD of serotonin: 50 nM | Metabolic  disorders | Pre-clinical stage with rabbit model;  Lack of long-term validation in large animal cohorts and human testing |
| MMNs-EGFET [S6] | ISF | AgNWs and ISMs | Na^+^, K^+^, Ca^2+^, and pH | Ca^2+^: 0.46 mA/decade, Na^+^: 3.43 mA/decade, pH: 0.31 mA/pH, and K^+^: 1.15 mA/decade;  LOD for Na^+^: 0.56 μM | Electrolytes monitoring | Self-powered by a triboelectric nanogenerator;  Lack of long-term validation |
| Wearable Aptalyzer [S7] | ISF | MB-labeled aptamer | Glucose and lactate | Glucose: 2.4 mM  Lactate: 1.04 mM | Diabetes | Aptamer-based biosensor;  Lack of system integration and human testing |
| Microneedle-based wearable sensor [S8] | ISF | Electrodeposited PPD | Lactate, alcohol, and glucose | - | Multi-event activities | Fully integrated system, on-body human testing;  Lack of efficient data analysis |
| eMPatch (this work) | ISF | Electrodeposited PEDOT:PSS and PB | Glucose, UA, cholesterol, Na^+^, K^+^, and pH | Glucose: 0.1122 μA cm^-2^ mM^-1^, UA: 0.001395 μA cm^-2^ μM^-1^, and cholesterol: 0.1637 μA cm^-2^ mM^-1^ | Metabolic disorders | Modularly-assembled flexible patch, ML-driven data analysis;  High power consumption, limited cohort size, lack of human testing, |

MIP, molecularly imprinted polymer; NiHCF, nickel hexacyanoferrate; PB, Prussian Blue; AgNWs, Silver nanowires; ISM, ion-selective membranes, MB, Methylene blue; PPD, poly-o-phenylenediamine; UA, uric acid; ML, machine learning.

**Supplementary References**

1. B. Zhong, X. Qin, H. Xu, L. Liu, L. Li et al., Interindividual- and blood-correlated sweat phenylalanine multimodal analytical biochips for tracking exercise metabolism. Nat. Commun. **15**(1), 624 (2024). <https://doi.org/10.1038/s41467-024-44751-z>
2. M. Wang, C. Ye, Y. Yang, D. Mukasa, C. Wang et al., Printable molecule-selective core-shell nanoparticles for wearable and implantable sensing. Nat. Mater. **24**, 589-598 (2025). <https://doi.org/10.1038/s41563-024-02096-4>
3. W. Park, H. Seo, J. Kim, Y.-M. Hong, H. Song, B. J. Joo et al., In-depth correlation analysis between tear glucose and blood glucose using a wireless smart contact lens. Nat. Commun. **15**(1), 2828 (2024). <https://doi.org/10.1038/s41467-024-47123-9>
4. R. T. Arwani, S. C. L. Tan, A. Sundarapandi, W. P. Goh, Y. Liu at al., Stretchable ionic–electronic bilayer hydrogel electronics enable in situ detection of solid-state epidermal biomarkers. Nat. Mater. **23**, 1115-1122 (2024). <https://doi.org/10.1038/s41563-024-01918-9>
5. J. Min, Ahn, H., Lukas, H., Gao, W. Continuous biochemical profiling of the gastrointestinal tract using an integrated smart capsule. Nat. Electron. **8**, 844-855 (2025). <https://doi.org/10.1038/s41928-025-01407-0>
6. R. Omar, M. Yuan, J. Wang, M. Sublaban, W. Saliba, Y. Zheng et al., Self-powered freestanding multifunctional microneedle-based extended gate device for personalized health monitoring. Sens. Actuators B Chem. **398**, 134788 (2024). <https://doi.org/10.1016/j.snb.2023.134788>
7. F. Bakhshandeh, H. Zheng, N. G. Barra, S. Sadeghzadeh, I. Ausri et al., Wearable aptalyzer integrates microneedle and electrochemical sensing for in vivo monitoring of glucose and lactate in live animals. Adv. Mater. e2313743 (2024). <https://doi.org/10.1002/adma.202313743>
8. F. Tehrani, H. Teymourian, B. Wuerstle, J. Kavner, R. Patel et al., An integrated wearable microneedle array for the continuous monitoring of multiple biomarkers in interstitial fluid. Nat. Biomed. Eng. **6**, 1214-1224 (2022). <https://doi.org/10.1038/s41551-022-00887-1>
